# Novel Endophytic Fungi from *Euchresta tubulosa* Dunn: Characterization of Their Bioactive Secondary Metabolites and Extracellular Enzymes

**DOI:** 10.3390/microorganisms14030664

**Published:** 2026-03-15

**Authors:** Xinlian Yin, Wei Guo, Qing Wang, Rushuang Nie, Dujiang Qing, Yao Hu, Sisi Hu, Linxin Wang, Xiaolin Ye, Shufeng Yao, Jiang Cheng

**Affiliations:** 1 School of Pharmaceutical Sciences, Jishou University, Jishou 416000, China; 2 Key Laboratory of Medicinal Resources Chemistry and Pharmacology in Wuling Mountainous of Hunan Province College, Jishou University, Jishou 416000, China

**Keywords:** *Euchresta tubulosa*, endophytic fungi, phylogenetic analysis, secondary metabolites, extracellular enzyme activity

## Abstract

The endangered ethnomedicinal plant *Euchresta tubulosa* harbors a valuable community of endophytic fungi, demonstrating significant potential for biotechnological applications. Endophytic fungi were isolated from *E. tubulosa* and identified to characterize their secondary metabolites and extracellular enzyme activities. Endophytic fungi were isolated from *E. tubulosa* using tissue explant culture and identified by morphological and molecular (ITS) analysis. The chemical profiles of strain fermentation products were analyzed by LC–MS/MS, while extracellular enzyme production (cellulase, protease, amylase) was assessed through chromogenic plate assays and liquid fermentation. The results indicated that a total of 55 endophytic fungi were isolated from *E. tubulosa*, assigned to 17 genera. Among these, three genera (*Colletotrichum*, *Fusarium*, and *Penicillium*) constituted the dominant groups, while four strains (including three novel species) represented potential new taxa. LC–MS/MS analysis revealed that fermentation products of the three novel endophytic fungal species contained bioactive compounds such as flavonoids and alkaloids; furthermore, bioactivity assays indicate that they exhibited significant degrees of antibacterial and antioxidant activity. Extracellular enzyme assays demonstrated that three *E. tubulosa*-derived endophytic strains exhibited multi-hydrolytic enzyme production capabilities. Notably, strain ETG-1-2-1 showed the highest amylase and cellulase activities (10.95 U/mL and 9.68 U/mL, respectively), while strain ETXG-1-1-1 displayed the highest protease activity (2.34 U/mL). This study provides the first systematic report on the diversity of endophytic fungi in *E. tubulosa*, their secondary metabolite profiles, and extracellular enzyme activities, establishing a theoretical foundation for discovering novel bioactive compounds and developing microbial resources, while also highlighting their ecological roles and biotechnological potential.

## 1. Introduction

The discovery of natural product-based drugs has long relied on wild plant resources. However, with growing challenges such as the depletion of these wild resources, identifying new biological sources has become an urgent need in contemporary drug development and related societal issues [1]. Endophytic fungi are a group of fungi that reside either permanently or temporarily within plant tissues (such as roots, stems, leaves, seeds, etc.), forming a symbiotic relationship with the host plant without causing overt disease symptoms [2,3,4]. In 1993, Stierle et al. isolated an endophytic fungus (*Taxomyces andreanae*) that produces paclitaxel from the phloem of the Pacific yew (*Taxus brevifolia* Nutt) [5]. This landmark discovery markedly expanded scientific understanding of the functional roles of plant endophytes.

Endophytic fungi interact with plants to shape community diversity and host specificity [6,7]. They are intricately linked to host growth and development [8], reproduction [9], repair mechanisms [10], and the secretion of metabolites [11], and are thus viewed as extensions of the host’s genetic repertoire [12]. Research by Taheri et al. revealed that endophytic fungal isolates from hairy vetch (*Vicia villosa* Roth) exhibit the potential to produce indole-3-acetic acid (IAA) and siderophores, along with phosphate- and potassium-solubilizing activities [13]. Among these, the newly described endophytic fungus *Penicillium griseofulvum* AKL25 demonstrated particularly high plant growth-promoting activity and shows promise as a microbial inoculant for enhancing the growth of hairy vetch and soybean in sustainable agriculture systems. In addition, endophytic fungi exhibit significant advantages in terms of metabolite diversity due to their rapid, reproducible, and unrestricted production, which is not affected by weather or seasonal factors [14]. Furthermore, their fermentation products exhibit diverse biological activities, including anticancer, antioxidant, and pathogen-inhibiting effects [15]. For example, a study by Sunil K. Deshmukh et al. pointed out that among the secondary metabolites produced by mangrove fungi, 91, 42, and 33 compounds have been reported to exhibit antibacterial, antifungal, and antiviral activities, respectively [16]. Separately, Zhang Yi et al. isolated endophytic fungi from *Carpesium abrotanoides* that demonstrated inhibitory activity against Hep G2 human liver cancer cells [17]. Among these, strain TMJ-54 exhibited the strongest suppression and significantly induced apoptosis. This finding highlights the potential of these fungi as valuable microbial resources for novel anti-hepatoma drug discovery. The above studies not only confirm the essentiality of endophytic fungi in host survival but also provide a basis for developing novel natural drug lead molecules from endophytic fungi to alleviate the shortage of plant-derived metabolites.

Endophytic fungi can also resist pathogenic bacteria by synthesizing extracellular enzymes, enhancing the host plant’s tolerance to biotic and abiotic stresses. Currently, *Trichoderma* species are widely deployed as biocontrol agents against soil-borne pathogens. Their secreted extracellular enzymes lyse pathogen cell walls, thereby achieving antimicrobial effects [18]. Moreover, compared to enzymes derived from animals and plants, microbial extracellular enzymes offer advantages in industrial production, including simpler manufacturing processes, lower costs, high stability, and specificity [19,20,21]. Therefore, their application in fields such as medicine, agriculture, food, and biofuels holds promising potential [22]. Hawar’s research revealed that endophytic fungi from Ziziphus spina-christi (Nabq) leaves, notably *Aspergillus niger* and *Cladosporium* sp., produce lipase and laccase [13]. Furthermore, the *Cladosporium* sp. exhibited high production of protease and pectinase. These enzymatic capabilities highlight the significant potential of endophytic fungi in biocontrol and clinical microbiology therapeutics.

*Euchresta tubulosa* Dunn, commonly known as Yapianqi, E-dougen, or Hudoulian, is a plant of the genus *Euchresta*. Recognized as a “prestigious ethnic medicine” in Tujia tradition, this plant is noted in *Hunan Medicinal Flora* for the use of its roots in treating gastric pain, abdominal discomfort, and sore throat [23]. *E. tubulosa* contains bioactive compounds including alkaloids, flavonoids, and terpenoids, which exhibit antitumor, antiviral, and anti-inflammatory properties, indicating significant pharmaceutical potential [24,25]. It is sporadically distributed in mid-subtropical evergreen broad-leaved forests along valleys and streams at elevations between 600 and 900 m in certain regions of Hunan, Guangdong, Guangxi, and Sichuan. This species thrives in shaded, moist, and humus-rich soils [18]. Due to its specific habitat requirements, large-scale cultivation is unfeasible. Additionally, the plant exhibits strong self-incompatibility, resulting in low natural fruiting and seed germination rates. As a consequence, its natural distribution has become extremely scarce, pushing it to the brink of extinction. It has been listed as a National Grade II Protected Plant [26]. The scarcity of medicinal material severely constrains research on its active constituents and pharmacological mechanisms, representing a major limiting factor for further exploitation.

Current research on *Euchresta tubulosa* Dunn primarily focuses on its chemical constituents, pharmacological activities, and clinical applications [27,28,29]. Internationally, reports on endophytic fungi isolated from *E. tubulosa* remain scarce. To better explore its endophytic microbial resources and discover structurally novel bioactive secondary metabolites and highly active enzymes, this study focused on the isolation and identification of endophytic fungi from *E. tubulosa*. Three distinctive strains were further investigated for their extracellular enzymes and secondary metabolites. This research aims to obtain functionally robust microbial isolates, thereby providing a theoretical foundation for future applications in biological control, novel drug development, and fermentation industries.

## 2. Materials and Methods

### 2.1. Biological Materials

Fresh *Euchresta tubulosa* Dunn plants were collected from Tianping Mountain (29°40′ N, 109°40′ E), Sangzhi County, Hunan Province, China on 13 July 2023 and 13 October 2023. Taxonomic identification was performed by Prof. Aiwen Dong. The plant materials were refrigerated at 4 °C and used for the isolation of endophytic fungi within 72 h. The strains used in the antimicrobial assay were *Escherichia coli* CMCC(B) 44102, *Staphylococcus aureus* CMCC(B) 26003, and *Candida albicans* CMCC(F) 98001, obtained from the Shanghai Bioresource Collection Center.

### 2.2. Main Reagents and Drugs

All chemicals and reagents were purchased from commercial suppliers: ethyl acetate, ethanol, and methanol from Concord Technology Co., Ltd. (Tianjin, China); sodium hypochlorite and anhydrous sodium carbonate from Tianjin Guangfu Technology Development Co., Ltd. (Tianjin, China); trichloroacetic acid, soluble starch, Congo red, and carboxymethylcellulose sodium from Tianjin Comio Chemical Reagent Co., Ltd. (Tianjin, China); casein, L-tyrosine, and Folin–Ciocalteu reagent from Ron Reagent Co., Ltd. (Shanghai, China); DNS reagent from Shanghai Yuanye Bio-Technology Co., Ltd. (Shanghai, China); Penicillin G sodium salt and kanamycin from Fuzhou Feijing Biotechnology Co., Ltd. (Fuzhou, China); lactophenol cotton blue staining solution from Qingdao Haibo Biotechnology Co., Ltd. (Qingdao, China).

Potato Dextrose Agar (PDA) and Potato Dextrose Broth (PDB) for fungal isolation and cultivation were obtained from Hope Bio-Technology Co., Ltd. (Qingdao, China).

### 2.3. Main Instruments

The following instruments were utilized: constant-temperature water bath (DZKW-D-6, Beijing Yongguangming Medical Instrument, Beijing, China); full-wavelength microplate reader (K6600A, Beijing Kaiwo Technology, Beijing, China); vertical autoclave (YXQ-LS-50SII, Shanghai Boxun Medical Equipment, Shanghai, China); biological microscope (PX43 FS6, Motic, Xiamen, China); constant-temperature shaker incubator (ZQPL-200, Tianjin Laiboterui Instrument, Tianjin, China); circulating water vacuum pump (SHB-III, Zhengzhou Great Wall Sci. & Tech., Zhengzhou, China); UV–Vis spectrophotometer (UV2400, Shanghai Shunyu Hengping Scientific Instrument, Shanghai, China); ultrapure water system (SMART-N30VF, Shanghai Kanglei Analytical Instrument, Shanghai, China); analytical balance (FA2004, Shanghai Shunyu Hengping Scientific Instrument, Shanghai, China); centrifuge (H1850R, Hunan Xiangyi Laboratory Instrument, Changsha, China); and rotary evaporator (RE-2000A, Shanghai Yarong Biochemical Instrument, Shanghai, China); biochemical incubator (BSP-400, Shanghai Boxun Medical Biological Instrument, Shanghai, China).

### 2.4. Preparation of Medium

#### Preparation of Solid Media

The media used in this study included both solid and liquid formulations. Solid media comprised PDA medium, inorganic salt starch medium, sodium carboxymethylcellulose (CMC-Na) medium, skim milk medium, and nutrient agar. Liquid media included PDB liquid medium, CMC-Na liquid medium, and inorganic salt starch and skim milk mixed liquid medium. Detailed compositions, preparation procedures, and sterilization conditions for all media are provided in Table S1.

### 2.5. Isolation of Endophytic Fungi from Euchresta tubulosa

Endophytic fungi isolated from *Euchresta tubulosa* fresh roots, stems, and leaves were placed firmly onto PDA (Potato Dextrose Agar) using the conventional tissue isolation method [30] and incubated for 3–7 days at 28 °C: hyphal growth around the tissue pieces was observed and recorded.

### 2.6. Identification and of Endophytic Fungi from Euchresta tubulosa

Colony Morphology Identification: A small amount of endophytic fungal strains preserved on PDA slant medium was streaked onto plate medium for activation (1–2 passages). Then, a 0.5 mm diameter fungal plug was inoculated onto PDA medium and incubated at 28 °C for 7–14 days. Growth characteristics including colony size, color, texture, surface patterns, margin morphology, presence of exudates, and odor were observed and recorded.

Morphological Characterization of Hyphae and Spores: Microscopic slides were prepared using the adhesive tape method [31], and the morphological examination of hyphae, conidiophores, spores, and other structures under a phase-contrast inverted microscope (model PX43 FS6).

The purified strains cultured of Identification were carried out at Chengdu Luoning Biotechnology Co., Ltd. Polymerase chain reaction (PCR) was used to amplify the internal transcribed spacer (ITS) regions 1 and 2 with the forward ITS1 5′-TCCGTAGGTGAACCTGCGG-3′and reverse ITS4 5′-TCCTCCGCTTATTGATATGC-3′PCR reaction system (50 μL): DNA template (20 ng/μL) 2 μL, forward and reverse primers (10 μmol/L) 2.5 μL each, 2 × PCR Bestaq Master Mix with dye 25 μL, ddH_2_O 18 μL. PCR reaction conditions: 95 °C for 5 min; 30 cycles of 94 °C for 45 s, 50 °C for 30 s, 72 °C for 45 s; 72 °C for 1 min. The PCR products were detected by 1% agarose gel electrophoresis. Concurrently, strain identification was performed by combining morphological characterization with molecular biological analysis the National Center for Biotechnology Information (NCBI) website (https://blast.ncbi.nlm.nih.gov/Blast.cgi, accessed 8 May 2025). Phylogenetic reconstruction was conducted using MEGA software (version 12.0) employing the Maximum Likelihood (ML) method with 1000 bootstrap replicates to assess branch support. This integrated approach enabled molecular-level identification of endophytic fungi from *Euchresta tubulosa* and determination of their taxonomic positions [32]. Endophytic fungi from *E. tubulosa* were taxonomically classified through molecular analysis when two or more strain sequences clustered within a monophyletic clade sharing ≥97.0–98.5% sequence similarity with reference strains at the species level. Classification thresholds were defined as: ≥90% similarity for genus-level identification, ≥85% for family-level, and ≥80% for order-level assignments [33].

### 2.7. Analysis of Fermentation Product Composition from Endophytic Fungi

Following established protocols [28], prepared the ethyl acetate extracted sample of the strain fermentation product and submitted to Novogene Co., Ltd. (Beijing, China) for compound identification via Liquid Chromatography–Tandem Mass Spectrometry (LC–MS/MS). Metabolites were characterized by comparing mass spectral data against public databases (GNPS, Pub-Chem, etc.).

### 2.8. Bioactivity Assay of Fermentation Products from Endophytic Fungi

#### 2.8.1. Antimicrobial Activity

Prepare samples with different concentration gradients: Antimicrobial concentration gradient: 20, 10, 8, 6, 4, 2, 1 mg/mL.Antioxidant concentration gradient: 4, 2, 1, 0.8, 0.6, 0.4, 0.2 mg/mL.

Preparation of bacterial suspensions: Cultivate *Escherichia coli*, *Staphylococcus aureus*, and *Candida albicans* separately in beef extract peptone broth and PDB broth. Select colonies with a concentration of approximately 1.0 × 10^6^ cfu/mL as the test bacterial suspensions. Antibacterial Activity Assay: Following the method of Wu Chunlin et al. [34], the critical value at which absorbance does not increase is used as the minimum inhibitory concentration (MIC) of the fermentation product.

#### 2.8.2. Antioxidant Activity

Following the method described by Yan Jun et al. [35], prepare solutions of Vc and fermentation products at different concentrations. Measure the absorbance values of A1, A2, and A3 at 510 nm, respectively. Calculate the clearance rate using the following formula:

(1) D = [(A1 − A2)/A3] × 100

where: A1 is the absorbance value without the fermentation product solution; A2 is the absorbance value with the fermentation product solution added; A3 is the absorbance value without salicylic acid.

Prepare the ABTS working solution according to the method described by Han Luoxia et al. [36] Take 100 μL each of the test solution, anhydrous ethanol, and Vc, and measure the absorbance values at 734 nm for OD_1_, OD_2_, and OD_3_, respectively. Calculate the scavenging rate using the following formula:

(2) D = [1 − (OD_1_ − OD_2_)/OD_0_] × 100

### 2.9. Evaluation of Extracellular Enzyme Activity in Endophytic Fungi Using the Clear Zone Method

Extracellular enzyme activity of endophytic fungi was evaluated using the clear zone method according to Shubha Jagannath et al. [37]. The diameters of the halo zones and fungal colonies were measured daily under specific staining conditions for each substrate:

• For skim milk agar, both the halo zones and colony diameters were measured directly.
• For starch-containing minimal salt agar, the plates were stained with dilute iodine solution to visualize halo zones, after which the diameters of the halo zones and fungal colonies were recorded.
• For CMC-Na agar, the plates were stained with Congo red reagent for 15 min, followed by destaining with NaCl solution for another 15 min. The presence of light-yellow halo zones was observed, and the diameters of the halo zones and fungal colonies were measured.

The enzyme activity zone (ZA) was calculated to assess the enzyme-producing capability of the endophytic fungi.

Enzyme activity was quantified using the enzymatic zone of activity (ZA) index [11], calculated as

(3) Enzymatic zone of activity (ZA) = Colony Diameter/Clear Zone Diameter

### 2.10. Extracellular Enzyme Activity Assays

#### 2.10.1. Preparation of Crude Enzyme Extracts

Crude enzyme extracts were prepared by aseptically transferring five 7-mm mycelial plugs from the actively growing margin of fungal colonies into 125 mL of liquid medium. Cultures were incubated in a rotary shaker (120 rpm) at 28 °C for 2–6 days. Following daily harvests from day 2 to day 6, the cultures underwent centrifugation at 10,000 r/min for 25 min at 4 °C, and the resultant supernatants served as crude enzyme extracts.

#### 2.10.2. Protease Activity Quantification via Folin–Ciocalteu Assay

A fresh casein substrate solution (200 μg/mL) was prepared following the method of Hu [30]. A series of tyrosine standard solutions with concentrations of 0, 10, 20, 30, 40, 50, 60, and 70 μg/mL was prepared by diluting the stock solution (200 μg/mL) with de-ionized water. The optical density (OD) values of these solutions were then measured according to the method described below. A standard curve was generated by plotting the OD values (ordinate) against the mass concentrations (abscissa), and a linear regression equation was established.

For enzyme activity determination, aliquots of casein solution and crude enzyme extract were pre-incubated separately at 40 °C for 10 min, followed by transfer of 1 mL reaction mixtures to 10-mL tubes. After 15 min incubation at 40 °C, reactions were terminated with 2 mL of 0.4 M trichloroacetic acid (TCA). Following 5 min sedimentation, samples were centrifuged at 3000× *g* for 10 min. Subsequently, 1 mL supernatant was combined with 5 mL 0.4 M Na_2_CO_3_ and 1 mL Folin–Ciocalteu (FC) reagent, incubated at 40 °C for 20 min, and absorbance measured at 680 nm. Control reactions received TCA prior to casein addition. Protease activity (U/mL) was calculated as:

(4) Activity = (OD_680_ × K × 4 × N)/(10 × V)

where K denotes tyrosine equivalence (μg) per unit absorbance (derived from standard curve), 4 represents total reaction volume (mL), N indicates dilution factor, 10 signifies reaction time (min), and V is crude enzyme volume (mL). One unit of protease activity was defined as the enzyme quantity liberating 1 μg tyrosine per minute under specified conditions.

#### 2.10.3. Quantification of Amylase and Carboxymethyl Cellulase (CMCase) Activities via DNS Assay

Fresh substrate solutions were prepared daily: 1% (*w*/*v*) starch by suspending 1.00 g in 50 mL boiling water with continuous stirring for 10 min before dilution to 100 mL; 1% (*w*/*v*) carboxymethyl cellulose sodium (CMC-Na) by dispersing 1.00 g onto boiling water followed by hydration and dilution to 100 mL.

A glucose stock solution (50 mg/mL) was prepared in deionized water. This stock was then diluted with deionized water to obtain a series of standard solutions with concentrations of 0, 100, 200, 300, 400, 500, 600, and 700 μg/mL. Each solution was then treated according to the procedure described below, and the optical density (OD) was measured.

A linear regression equation was derived by plotting the mass concentration (as abscissa) against the OD value (as ordinate). Detection method: Refer to the method of Hu [38], For enzymatic analysis, crude enzyme extract, phosphate buffer (0.05 mol/L, pH 7.0), and 1% substrate were pre-incubated at 37 °C for 10 min. Reaction mixtures containing 1 mL enzyme extract, 2 mL buffer, and 0.5 mL substrate were incubated at 37 °C for 10 min, terminated with 1 mL of 0.4 mol/L NaOH, supplemented with 100 μL DNS reagent, and heated in a boiling water bath for 6 min. After cooling and 10–20 min equilibration, absorbance was measured at 520 nm. Control reactions received NaOH prior to substrate addition. Carboxymethyl cellulase (CMCase) activity was determined identically using CMC-Na substrate. One enzyme unit (U) was defined as the amount catalyzing liberation of 1 μmol reducing sugar per minute under assay conditions. Extracellular enzyme activity (U/mL) was calculated as:

(5) *X* = (*c* × *V*_1_)/(*t* × *V*_2_)

where *c* represents reducing sugar concentration (μmol/mL, glucose-equivalent from standard curve), *V*_2_ denotes total reaction volume, *t* signifies reaction time (10 min), and *V*_1_ is crude enzyme volume.

#### 2.10.4. Standard Curves

A calibration curve was established by quantifying glucose standard solutions using the DNS assay, with absorbance measurements taken at 520 nm. The resulting calibration equation was y = 1.3143x − 0.00051 (R^2^ = 0.9986), as shown in Figure S17A. Similarly, a standard curve for tyrosine was generated using the Folin method, with absorbance measured at 680 nm, yielding the regression equation y = 9.84247x − 0.00608 (R^2^ = 0.99956) (Figure S17B).

## 3. Results

### 3.1. Isolation and Identification of Endophytic Fungi in the Euchresta tubulosa

**Figure 1:**
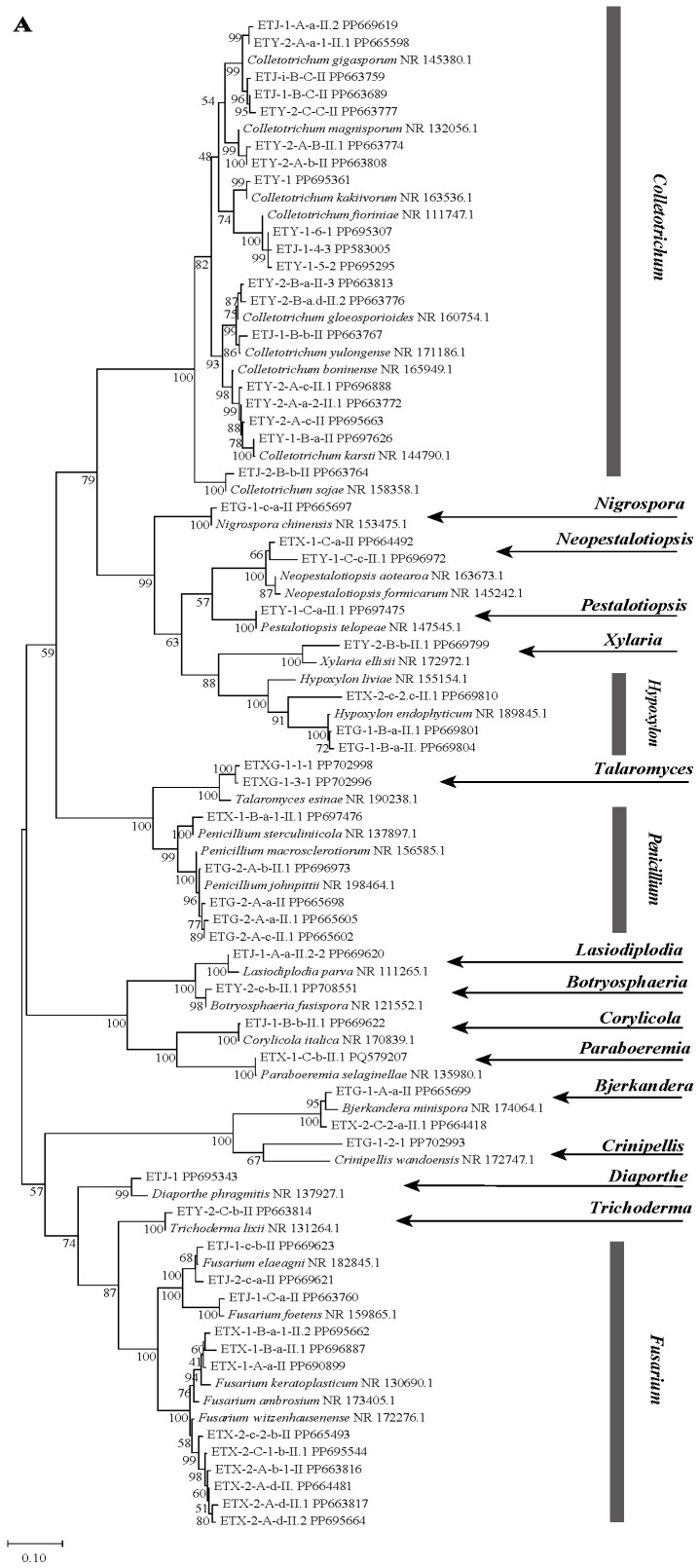

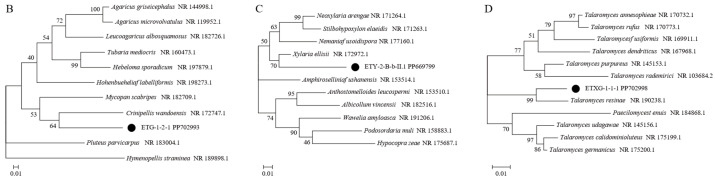
Phylogenetic trees based on ITS sequences of endophytic fungi from *Euchresta tubulosa* (**A**). Trees for novel taxa: ETG-1-2-1 ((**B**), novel genus), ETY-2-B-b-II.1 ((**C**), novel species in *Xylaria*), and ETXG-1-1-1 ((**D**), novel species in *Talaromyces*). Numbers at branches indicate bootstrap values (>50% shown). Scale bars represent sequence divergence. Branch lengths correspond to genetic distance. Letters/numbers following strain names denote GenBank accession numbers.

A total of 55 endophytic fungal strains were isolated from fresh *Euchresta tubulosa* plants. Based on preliminary rRNA-ITS sequence analysis, these strains were classified into 33 species and 17 genera, which were distributed across the following genera: *Fusarium*, *Penicillium*, *Pestalotiopsis*, *Xylaria*, *Trichoderma*, *Bjerkandera*, *Neopestalotiopsis*, *Botryosphaeria*, *Nigrospora*, *Lasiodiplodia*, *Corylicola*, *Hypoxylon*, *Talaromyces*, *Crinipellis*, *Diaporthe*, *Paraboeremia* and *Colletotrichum*. The dominant genera were *Colletotrichum* (34.5%), *Fusarium* (21.8%), and *Penicillium* (9.1%). The obtained ITS sequences were deposited in the GenBank database to acquire accession numbers. The most similar ITS region gene sequences were identified using NCBI-BLAST homology comparison, and a phylogenetic tree was constructed (Figure 1A).

NCBI-BLAST homology analysis revealed that strains ETG-1-2-1, ETY-2-B-b-II.1, ETXG-1-1-1 and ETXG-1-3-1 showed ≤97% sequence similarity to their closest relatives, indicating that these strains may represent novel taxa at the genus or species level [33]. Among them, the NCBI-BLAST homology alignment results showed that the sequence homology between strain ETG-1-2-1 and its most similar strain was not higher than 90%, leading to its identification as a novel genus. Strains ETY-2-B-b-II.1, ETXG-1-1-1, and ETXG-1-3-1 exhibited homology not exceeding 97% compared to their closest relatives, and were consequently identified as novel species. Notably, strains ETXG-1-1-1 and ETXG-1-3-1 matched the same reference sequence in the NCBI-BLAST alignment (Figures S14 and S15), and shared 99.46% sequence identity (Figure S16). Furthermore, Figure 1A demonstrated that the genetic distance between these two strains is minimal, identifying them as sister strains of a novel species within the genus *Talaromyces*.

Phylogenetic analysis based on ITS sequences placed all three endophytes (ETG-1-2-1, ETY-2-B-b-II.1, and ETXG-1-1-1) within distinct, well-supported clades (bootstrap values > 50%), as shown in Figure 1B–D. Specifically, ETG-1-2-1 was most closely related to *Crinipellis wandoensis* (NR 172747.1) with a bootstrap value of 64% and a BLAST alignment similarity of 85.28%, and was identified as a new genus of the family *Marasmiaceae*., It was named “*Neocrinipellis albifloccum* ETG-1-2-1” and deposited at the China General Microbiological Culture Collection Center with the accession number CGMCC No. 41962 [39]. Strain ETY-2-B-b-II.1 was most closely related to *Xylaria ellisii* (NR 172972.1) with a bootstrap value of 70% and a BLAST alignment similarity of 92.16%, and was identified as a new species of the genus *Xylaria*., It was named “*Xylaria cinereonitens* ETY-2-B-b-II.1” (CGMCC No. 42144) [40]. Strain ETXG-1-1-1 was most closely related to *Talaromyces resinae* (NR 190238.1) with a bootstrap value of 99% and exhibited 95.86% similarity in BLAST alignment, leading to its identification as a novel species within the genus *Talaromyces*. The strain was named “*Talaromyces polymorphus* ETXG-1-1-1” (CGMCC No. 41927) [41]. Furthermore, the three endophytic fungal strains isolated from *Euchresta tubulosa* were located in distinct clades in Figure 1A, indicating a distant phylogenetic relationship among them. The fungal sequencing results were submitted to the GenBank database, and the corresponding GenBank accession numbers for the tested strains were obtained, as summarized in Table 1.

### 3.2. Morphological Characterization of Selected Endophytic Fungi from Euchresta tubulosa

**Figure 2:**
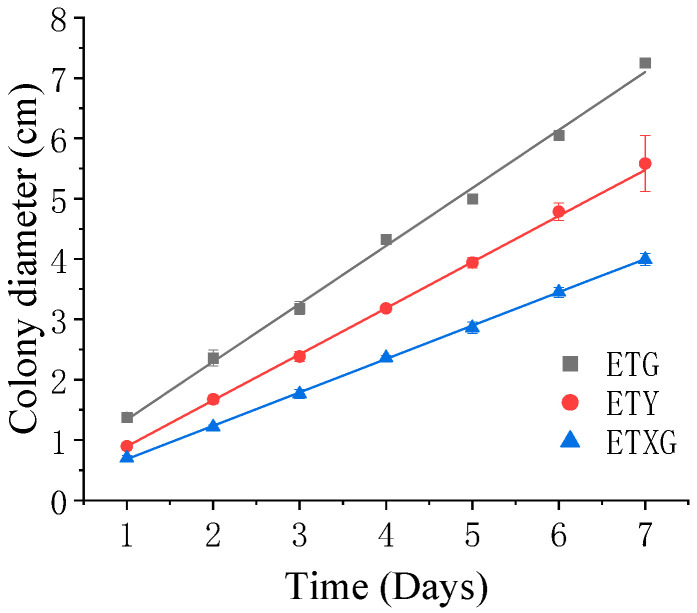
Growth dynamics of endophytic fungi from *Euchresta tubulosa* on PDA medium.

**Figure 3:**
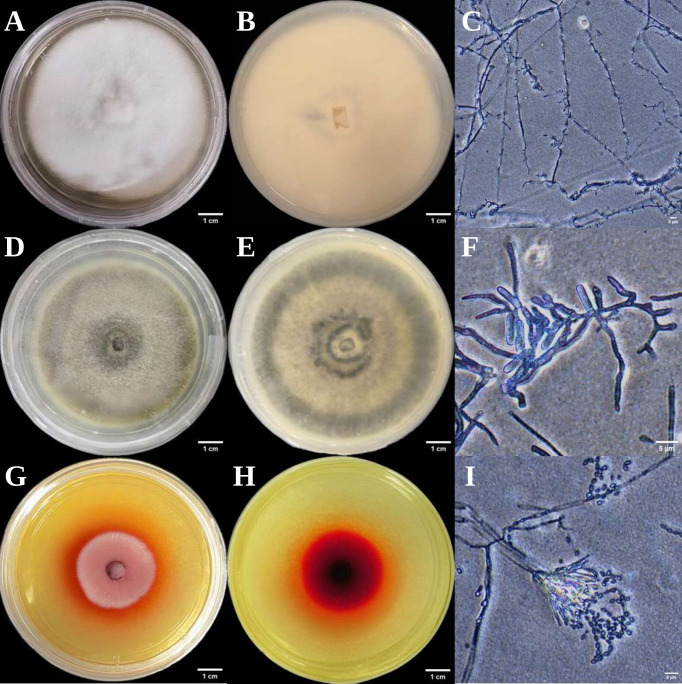
Colonial and micro-morphological features of three endophytic fungal strains isolated from *Euchresta tubulosa*. The obverse colony morphology (**A**), reverse colony morphology (**B**), and microscopic observation of mycelia (**C**) of strain ETG-1-2-1; the obverse colony morphology (**D**), reverse colony morphology (**E**), and microscopic observation of mycelia (**F**) of strain ETY-2-B-b-II.1; the obverse colony morphology (**G**), reverse colony morphology (**H**), and microscopic observation of mycelia (**I**) of strain ETXG-1-1-1.

The three endophytic fungi isolated from *Euchresta tubulosa* were morphologically identified based on their morphological characteristics (Table 2), and their growth on PDA medium was documented (Figure 2 and Figure 3). The strain ETG-1-2-1 grew radially from the inoculation point to form a circular colony with an even margin on PDA medium. It exhibited white, dense, and villous surface mycelia, secreted no soluble pigments, and reached a diameter of 7.25 cm at 7 d. The average radial growth rate was calculated to be (9.61 ± 0.26) mm/day. On PDA medium, colony ETY-2-B-b-II.1 was initially white and turned black upon maturation, exhibiting a thin velvety texture and a relatively slow growth rate. After 7 days of incubation, the colony reached a diameter of approximately 5.58 cm, with a growth rate of (7.64 ± 0.06) mm/day. The edge of the colony was entire, with a grayish-white sporulating structure observed in the center. Concentric rings were visible on the reverse side. Microscopic examination revealed slender and uniform hyphae with distinct branching. Conidia were regularly oval-shaped, partly attached to the tips of hyphal branches and partly dispersed as individual units. On PDA medium, ETXG-1-1-1 formed colonies with dense, velvety mycelia, exhibiting a flat center and radial wrinkles. The colony displayed a white peripheral zone and a red central region. Mature spores were abundant, and easily detached. The reverse side showed a radial dark red pigmentation that was most intense in the center and faded gradually toward the margin. The strain demonstrated a slow growth rate, reaching a diameter of approximately 3.99 cm after 7 days of incubation at 28 °C, with an average radial growth rate of 5.53 ± 0.06 mm/d. Hyphal examination under inverted phase-contrast microscope revealed septate and elongated filamentous structures. Conidia were solitary or aggregated in sporodochial clusters, varying in length. The conidia have a smooth surface, round or nearly circular, arranged in chains or scattered around the spore stalk.

### 3.3. LC–MS Analysis of Secondary Metabolites from Endophytic Fungi

To investigate their chemical profiles, we analyzed the secondary metabolites of three endophytic fungal strains derived from *Euchresta tubulosa* using liquid chromatography–mass spectrometry (LC–MS). Total ion chromatograms (TIC) in positive (POS) and negative (NEG) ionization modes (Figure S1) and mass spectra of selected metabolites (Figure S2) were acquired. Metabolite annotation was performed by matching against the mzCloud, mzVault, and MassList databases.

**Figure 4:**
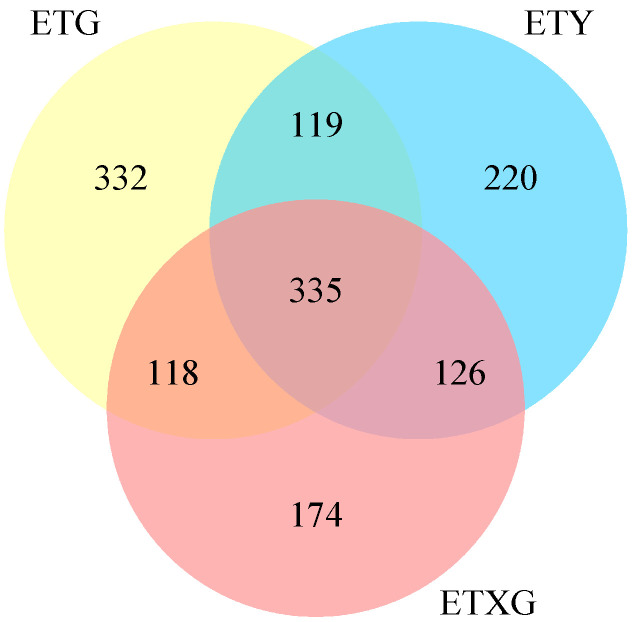
Venn diagram of secondary metabolites from three endophytic fungal strains of *Euchresta tubulosa*.

**Figure 5:**
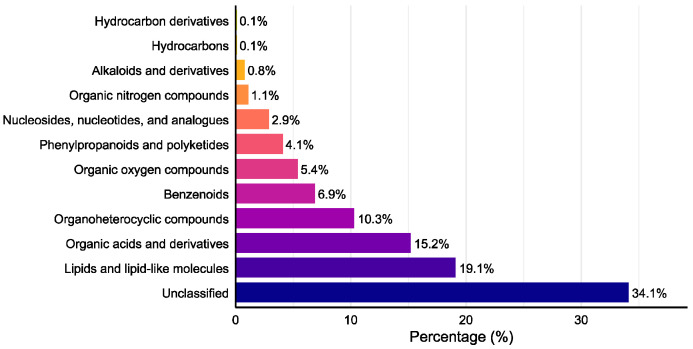
SuperClass distribution of detected secondary metabolites.

Venn diagram analysis (Figure 4) revealed 1424 total metabolites, with 698 shared compounds (49.0%) across all three strains. SuperClass classification (Figure 5) demonstrated that lipids and lipid-like molecules, organic acids and derivatives, organoheterocyclic compounds, and benzenoids represented the most abundant classes after unclassified metabolites. Additionally, we identified 11 alkaloids, 18 flavonoids, 26 terpenoids, and 59 steroids.

The partially active components from strains were organized into a table, where the strains were denoted as ETG, ETY, and ETXG, representing ETG-1-2, ETY-2-B-b-II.1, and ETXG-1-1-1, respectively (Table 3). Meanwhile, the mass spectra of these compounds are provided in the Supplementary Material, Figures S3–S11. These active components included reserpine, known for its significant antihypertensive, tranquilizing, and antipsychotic effects; trigonelline, reported to exhibit activities such as lowering blood glucose and improving blood lipids; and oxymatrine, which possesses anti-inflammatory, anti-fibrotic, and anti-tumor properties [22]. Notably, these classes of compounds were also detected in preliminary analyses of *Euchresta tubulosa* plant samples conducted by our research group using LC–MS/MS technology. Li Weixin [25] identified oxymatrine in the stems of *E. tubulosa* through spectroscopic methods such as NMR and MS. Additionally, Lei Jiaxin performed pseudo-targeted metabolomics analysis on *E. tubulosa* plants and detected 145 flavonoid compounds, among which glycitein, daidzein, naringenin, formononetin, genistein, kaempferol, sakuranetin, and hesperetin were also present in the secondary metabolites of the endophytic fungi studied here. These findings indicate similarities between the secondary metabolites of these three endophytic fungal strains and the active components of *E. tubulosa*, suggesting a potential association with the growth process of *E. tubulosa*.

### 3.4. Bioactivity Assay of Fermentation Products from Endophytic Fungi

#### 3.4.1. Antimicrobial Activity Assay

The minimum inhibitory concentrations (MICs) of ethyl acetate extracts derived from the fermentation broths of three endophytic fungi ETG-1-2-1, ETXG-1-1-1, and ETY-2-B-b-II.1 isolated from *Euchresta tubulosa* are summarized in Table 4 against *Escherichia coli*, *Staphylococcus aureus*, and *Candida albicans*. Among the tested extracts, that from strain ETG-1-2-1 exhibited the most potent activity against *E. coli*, with an MIC of 2.5 mg/mL. All three extracts displayed equivalent inhibitory efficacy against *S. aureus*, yielding identical MIC values of 2.5 mg/mL. Notably, the ETG-1-2-1-derived extract also demonstrated the lowest MIC (1.25 mg/mL) against *C. albicans*, indicating superior antifungal activity relative to the other two strains.

#### 3.4.2. Hydroxyl Radical Scavenging Capacity Assay

**Figure 6:**
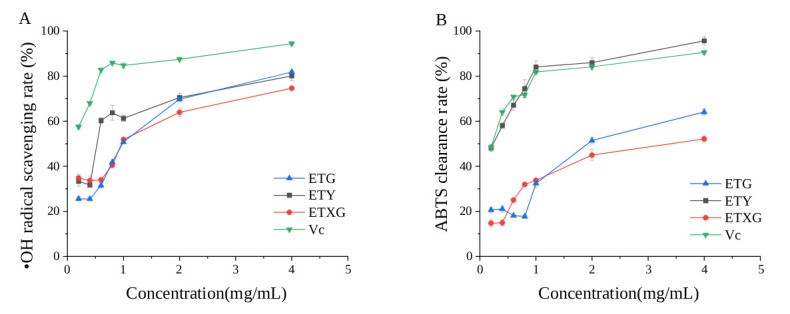
Scavenging rates of hydroxyl radicals (**A**) and ABTS radicals (**B**) by fermentation products from endophytic fungi of *Euchresta tubulosa* at different concentrations.

The hydroxyl radical scavenging capacities of ethyl acetate extracts derived from fermentation broths of three endophytic fungi ETG-1-2-1, ETXG-1-1-1, ETY-2-B-b-II.1, and vitamin C are presented in Figure 6A. As illustrated, the scavenging activities of the extracts from ETG-1-2-1, ETXG-1-1-1, and ETY-2-B-b-II.1 increased markedly within the concentration range of 0.4 to 1 mg/mL, followed by a marginal enhancement from 1 to 4 mg/mL. Notably, the ethyl acetate extracts of ETG-1-2-1 and ETY-2-B-b-II.1 exhibited superior scavenging rates of 81.84% and 80.10%, respectively, at 4 mg/mL. Conversely, ETXG-1-1-1 demonstrated a comparatively lower scavenging activity of 74.60%.

#### 3.4.3. ABTS Radical Scavenging Activity Assay

The ABTS radical scavenging capacity of ethyl acetate extracts derived from the fermentation broths of three endophytic fungi ETG-1-2-1, ETXG-1-1-1, and ETY-2-B-b-II.1 as well as that of ascorbic acid (Vc) is presented in Figure 6B. The results demonstrate that the ethyl acetate extract from the fermentation broth of strain ETY-2-B-b-II.1 exhibits exceptionally potent ABTS radical scavenging activity, achieving 95.69% scavenging at a concentration of 4 mg/mL—significantly surpassing the positive control (Vc) under identical experimental conditions. In contrast, the extracts from strains ETG-1-2-1 and ETXG-1-1-1 displayed markedly lower scavenging activities, with values of 52.14% and 64.07%, respectively.

### 3.5. The Extracellular Enzyme Activity of the Endophytic Fungi from Euchresta tubulosa Was Evaluated by Transparent Circle Method

#### 3.5.1. Protease Activity

**Figure 7:**
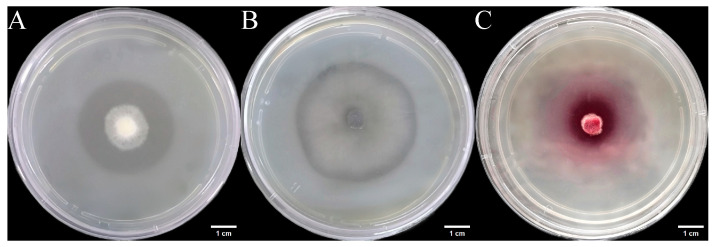
Detection of protease activity of endophytic fungi from *Euchresta tubulosa.* The incubation time of strains ETG−1−2−1 (**A**), ETY−2−B−b−II.1 (**B**) and ETXG−1−1−1 (**C**) was 4 d, 6 d and 5 d, respectively. The transparent area in the middle of the culture medium is the enzyme active zone, and the white area on the outer ring of the culture medium is the inactive zone. In addition, the deep red area in the middle of the culture medium in Figure C represents the enzyme activity zone.

**Figure 8:**
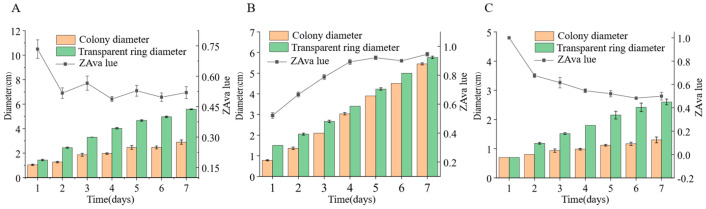
Comparison of protease activities in endophytic fungi: ETG−1−2−1 (**A**), ETY−2−B−b−II.1 (**B**) and ETXG−1−1−1 (**C**) from *Euchresta tubulosa*.

We inoculated three endophytic fungal strains isolated from *Euchresta tubulosa* onto skim milk agar plates. After 7-day incubation, proteolytic halos were observed (Figure 7). Halo and colony diameters were measured to calculate the enzymatic zone of activity (ZA), with results presented in dual-axis plots (Figure 8). All strains exhibited progressive increases in both colony and halo diameters, though growth kinetics differed temporally: ETG-1-2-1: Demonstrated significant ZA fluctuations during initial growth (days 1–2), stabilizing thereafter with consistently strong protease activity (ZA < 0.69 from day 2 onward). This indicates stable enzyme secretion proportional to biomass accumulation (Figure 8A). ETY-2-B-b-II.1: Displayed increasing ZA (declining enzyme activity) over time. Early-stage halo expansion exceeded colony growth, yielding strong activity (ZA < 0.69) during days 1–2 and moderate activity (ZA = 0.788) at day 3. Late-phase parallel growth of colony and halo suggested nutrient-dependent enzyme production linked to microbial proliferation (Figure 8B). ETXG-1-1-1: Showed decreasing ZA (enhancing enzyme activity) despite limited overall growth. Sustained high protease activity (ZA < 0.69 from day 2) indicated efficient catalytic stability despite low enzyme yield (Figure 8C).

#### 3.5.2. Amylase Activity

**Figure 9:**
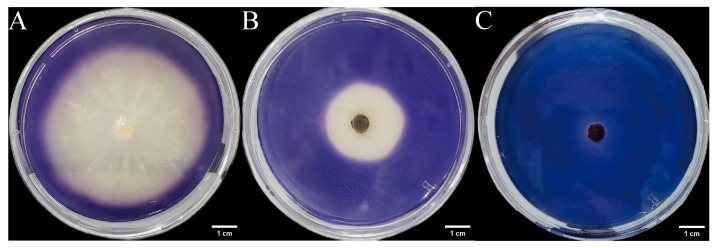
Amylase activity of endophytic fungi isolated from *Euchresta tubulosa.* The incubation time of strains ETG−1−2−1 (**A**), ETY−2−B−b−II.1 (**B**) and ETXG−1−1−1 (**C**) was 7 d, 5 d and 5 d, respectively. The transparent central area of the culture medium represents the enzymatically active zone, while the blue outer ring indicates the inactive zone.

**Figure 10:**
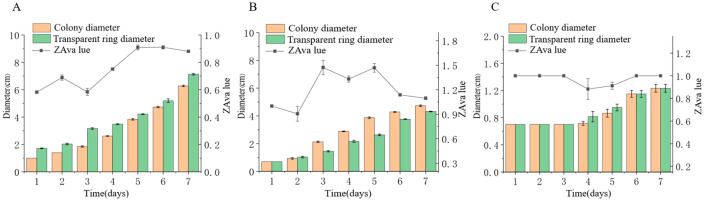
Comparison of amylase activities in endophytic fungi: ETG−1−2−1 (**A**), ETY−2−B−b−II.1 (**B**) and ETXG−1−1−1 (**C**) from *Euchresta tubulosa*.

Three endophytic fungal strains isolated from *Euchresta tubulosa* were inoculated onto mineral-starch agar plates. After 7-day incubation, iodine staining revealed clear zones (Figure 9) Colony and clear zone diameters were measured to calculate hydrolytic coefficients (ZA), with results plotted on dual-axis graphs (Figure 10). Strain ETG-1-2-1 exhibited slow initial colony growth but rapid clear zone expansion during days 1–3 (ZA < 0.69), indicating strong amylase activity and a lag phase for environmental adaptation; subsequent colony acceleration coincided with fluctuating ZA, reflecting stage-dependent variations in exoenzyme efficiency (Figure 10A). Strain ETY-2-B-b-II.1 showed sustained increases in both parameters, but colony diameters exceeded clear zones from day 3 onward (ZA > 1), precluding enzymatic assessment by this metric—potentially due to efficient utilization of hydrolytic products and rapid growth (Figure 10B). Strain ETXG-1-1-1 demonstrated negligible growth until day 4 with minimal expansion (ZA > 0.85 throughout), suggesting limited amylase production, weak activity, and inadequate substrate degradation under these nutritional conditions (Figure 10C).

#### 3.5.3. Carboxymethyl Cellulase (CMCase) Activity

**Figure 11:**
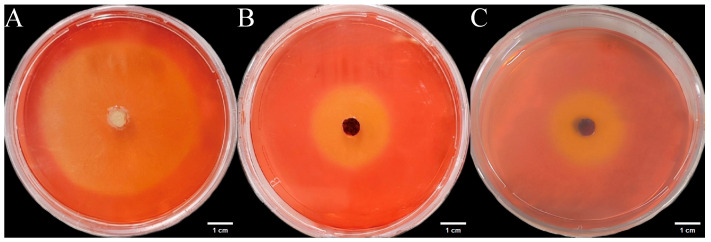
Detection of CMCase activity in endophytic fungi from *Euchresta tubulosa.* The incubation time of strains ETG−1−2−1 (**A**), ETY−2−B−b−II.1 (**B**) and ETXG−1−1−1 (**C**) was 7 d, 5 d and 4 d, respectively. The light yellow central area of the culture medium is the enzymatically active zone, while the red outer ring is the inactive zone.

**Figure 12:**
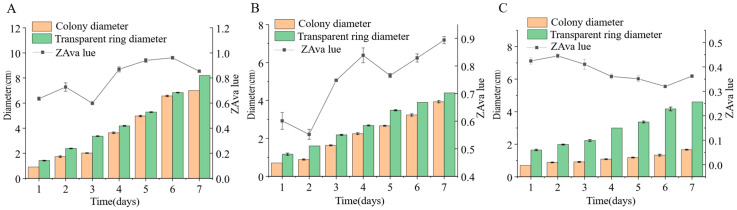
Comparison of CMCase activities in endophytic fungi: ETG−1−2−1 (**A**), ETY−2−B−b−II.1 (**B**) and ETXG−1−1−1 (**C**). from *Euchresta tubulosa*.

We inoculated three endophytic fungi derived from *Euchresta tubulosa* onto carboxymethyl cellulose sodium (CMC-Na) agar plates. After 7 days of culture, Congo red staining and NaCl destaining revealed hydrolysis zones (Figure 11). Colony diameters (CD) and hydrolysis zone diameters (HZD) were measured to calculate enzymatic activity zones (ZA = CD/HZD), with photographic documentation in Figure 12. Strain ETG-1-2-1 exhibited characteristic growth phases: lag, exponential, and stationary. Significant ZA fluctuations occurred during cultivation, particularly in the initial 48 h, indicating physiological instability during adaptation to the novel environment through enzymatic and transport system restructuring. Optimal ZA (0.599) occurred at day 3 (Figure 12A). Strain ETY-2-B-b-II.1 showed progressive increases in both CD and HZD throughout cultivation. However, its ZA profile displayed biphasic fluctuations: an initial decrease followed by recovery. This pattern suggests: early phase (days 1–2): rapid carboxymethyl cellulase (CMCase) synthesis facilitated carbon acquisition, triggering exponential growth. Mid-late phase (>day 4): Nutrient limitation induced secondary enzyme induction, though metabolic waste accumulation ultimately suppressed both enzymatic activity and growth rate. Minimum ZA (0.552, optimal value) was recorded at day 2 (Figure 12B). Strain ETXG-1-1-1 demonstrated severely restricted growth on CMC-Na agar plates: 73.68% reduction in growth rate compared with PDA controls CD expansion significantly lagged behind other strains. Conversely, HZD progressively increased (notably during days 2–4), driving accelerated ZA decline (0.44530→0.41073→0.36111). Critically, ZA consistently remained within the high-activity range (0.30–0.45), confirming this strain’s exceptional capacity for sustained CMCase hyperproduction and stability (Figure 12C).

### 3.6. Extracellular Enzyme Activity

#### 3.6.1. Extracellular Protease Activity

**Figure 13:**
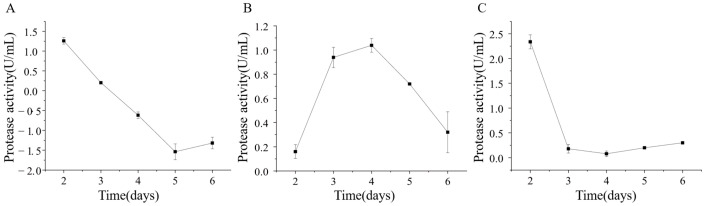
Changes of extracellular protease activity of three endophytic fungi: ETG−1−2−1 (**A**), ETY−2−B−b−II.1 (**B**) and ETXG−1−1−1 (**C**). from *Euchresta tubulosa*.

The extracellular protease production of strains ETG-1-2-1, ETY-2-B-b-II.1, and ETXG-1-1-1 was evaluated by fermentation in a skim milk-based liquid medium. Crude enzyme extracts were collected at various time points and subsequently assayed for protease activity, as summarized in Figure 13 The protease activity of strain ETG-1-2-1 reached its highest value of 1.26 ± 0.08 U/mL on day 2 of culture, after which it began to decline until the viability was undetectable. The protease activity of strain ETY-2-B-b-II.1 increased first and then decreased, reaching a peak of 1.04 ± 0.06 U/mL on the 4th day. The protease activity of strain ETXG-1-1-1 had a maximum value of 2.34 ± 0.14 U/mL on the second day, followed by a sharp decrease on the third day, and then a slight upward trend.

Analysis revealed that the extracellular protease activity in strains ETG-1-2-1 and ETXG-1-1-1 primarily occurred during the early cultivation period. Based on the mycelial growth throughout the liquid culture process, it is proposed that the nutrient profile of the skim milk-based medium strongly supported initial fungal proliferation, promoting rapid and vigorous growth that facilitated high levels of enzyme synthesis. In contrast, the peak enzyme production of strain ETY-2-B-b-II.1 exhibited delayed peak enzyme production, possibly due to differences in temporal nutrient utilization or distinct physiological regulation mechanisms.

#### 3.6.2. Extracellular Amylase Activity

**Figure 14:**
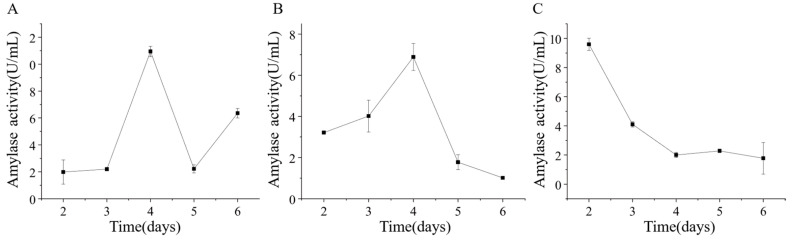
Changes of extracellular amylase activity of three endophytic fungi: ETG−1−2−1 (**A**), ETY−2−B−b−II.1 (**B**) and ETXG−1−1−1 (**C**). from *Euchresta tubulosa*.

Strains ETG-1-2-1, ETY-2-B-b-II.1, and ETXG-1-1-1 were cultured in mineral-starch broth to assess extracellular amylase production. Crude enzyme extracts collected at various time points exhibited distinct amylolytic activity profiles, as shown in Figure 14 ETG-1-2-1 exhibited gradual activity increase from day 2, surged sharply during days 3–4 (peak: 10.95 ± 0.38 U/mL on day 4), followed by rapid decline and a secondary increase on day 6 (6.35 ± 0.35 U/mL); ETY-2-B-b-II.1 showed unimodal activity peaking at 6.89 ± 0.66 U/mL on day 4; whereas ETXG-1-1-1 reached maximal activity (9.60 ± 0.42 U/mL) on day 2, followed by rapid decline.

#### 3.6.3. Extracellular Carboxymethyl Cellulase (CMCase) Activity

**Figure 15:**
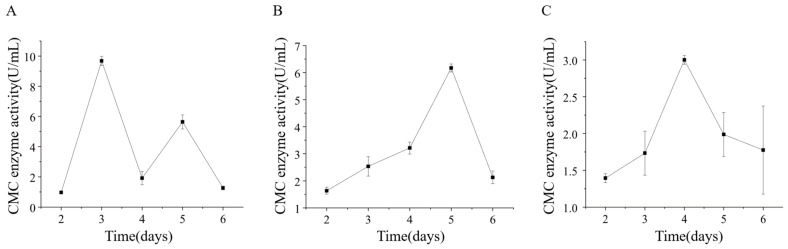
Changes of extracellular carboxymethyl cellulase (CMCase) activity of three endophytic fungi: ETG−1−2−1 (**A**), ETY−2−B−b−II.1 (**B**) and ETXG−1−1−1 (**C**). from *Euchresta tubulosa*.

Strains ETG-1-2-1, ETY-2-B-b-II.1, and ETXG-1-1-1 were cultivated in sodium carboxymethyl cellulose (CMC-Na) liquid medium under submerged fermentation (SmF) conditions to evaluate extracellular cellulase production. Crude enzyme extracts collected at various time points were assayed for carboxymethyl cellulase (CMCase) activity, as presented in Figure 15 ETG-1-2-1 reached its peak CMCase activity (9.68 ± 0.30 U/mL) on day 3, followed by a sharp decline and a subsequent rebound to 5.64 ± 0.47 U/mL by day 5. Both ETXG-1-1-1 and ETY-2-B-b-II.1 exhibited unimodal activity profiles: ETY-2-B-b-II.1 achieved maximum activity (6.17 ± 0.15 U/mL) on day 5, while ETXG-1-1-1 peaked earlier (3.00 ± 0.06 U/mL) on day 4.

ETG-1-2-1 demonstrated a bimodal carboxymethyl cellulase (CMCase) production pattern. According to Cao Chunlei et al. [15], this phenomenon may stem from the strain’s limited cellulose utilization capacity. During early cultivation, mycelia prioritize nucleic acid and protein synthesis for subsequent growth, triggering initial CMCase induction. Subsequent rapid mycelial expansion depletes intracellular amino acid pools, impairing CMCase synthesis. As readily metabolizable glucose diminishes in the medium, secondary CMCase secretion is induced until nutrient exhaustion ultimately arrests growth.

## 4. Discussion

Owing to the long-term co-evolution of endophytic fungi with host plants, these fungi engage in synergistic interactions that enhance the host’s adaptive responses to biotic and abiotic stresses. This mutual adaptation has led to the establishment of unique, host-specific endophytic assemblages [3]. A total of 55 endophytic fungal strains were isolated from different tissues of *Euchresta tubulosa*, comprising 18 from leaves, 12 from stems, and 25 from roots. The morphological typing method based on macro-morphological characteristics is insufficient to fully reflect the phylogenetic relationships of endophytic fungi, especially for non-sporulating fungal species, which presents significant limitations. The ITS region is currently the standard DNA marker for fungal identification [42,43]. Based on combined morphological and molecular analyses, these isolates were assigned to 17 genera, with *Colletotrichum*, *Fusarium*, and *Penicillium* identified as the predominant genera. The detected ITS sequences were subjected to NCBI-BLAST alignment, with the following criteria applied for taxonomic identification: a similarity of ≥97.0–98.5% corresponds to species-level identification, ≥90% to genus-level identification, ≥85% to family-level identification, and ≥80% to order-level identification [33]. Based on these criteria, strain ETG-1-2-1 belonged to a novel genus within the family *Marasmiaceae*, while strains ETY-2-B-b-II.1 and ETXG-1-1-1 were identified as novel species belonging to the genera *Xylaria* and *Talaromyces*, respectively.

Endophytic fungi constitute a major reservoir of unexplored fungal diversity and are capable of producing a vast array of diverse secondary metabolites [44]. They have garnered significant scientific attention due to their potential as novel biological sources of natural active compounds, their role in mediating intraspecific and interspecific signaling, and their ability to alleviate abiotic and biotic stresses in plants [25,45]. Secondary metabolites from three endophytic fungal strains isolated from different tissues of *Euchresta tubulosa*, were analyzed using LC–MS/MS. Chemical profiling revealed a diverse spectrum of metabolites, including alkaloids, flavonoids, terpenoids, stilbenes, tetracyclines, and steroids. Consistent with previous reports, endophytic fungi from plants not only biosynthesized compounds identical or analogous to those of their host medicinal plant but also produced structurally novel metabolites with putative bioactivities [25]. Notably, strains ETG-1-2-1 and ETXG-1-1-1 were found to produce oxymatrine, a compound also present in *E. tubulosa*, which exhibits broad pharmacological properties such as anti-inflammatory, antiviral, antifibrotic, and antitumor effects [18]. Li Danyang et al. reported that alkaloid compounds primarily exert antibacterial effects by disrupting key structural components of microbial cells, including the cell wall and cytoplasmic membrane [46]. Building upon this finding, Liu Mengxiao et al. demonstrated that oxymatrine significantly inhibits fungal growth through multiple mechanisms: suppression of biofilm formation, impairment of fungal cell membrane integrity, and reduction of conidial adhesion capacity [47]. Fermentation products of these strains demonstrated significant inhibitory activity against both *Staphylococcus aureus* and *Bacillus subtilis*. Against this background, the present study evaluated the antibacterial activity of acetone-soluble extracts derived from the fermentation broths of three endophytic fungal strains isolated from *E. tubulosa*. The results revealed varying degrees of inhibitory activity against *Escherichia coli*, *Staphylococcus aureus*, and *Candida albicans*, with strain ETG-1-2-1 exhibiting the strongest antimicrobial effect. Supporting this observation, previous studies have shown that oxymatrine, purified from the rhizomes of *E. tubulosa*, displays potent antibacterial activity against both *S. aureus* and *E. coli*, as evidenced by low minimum inhibitory concentrations (MICs) and minimum bactericidal concentrations (MBCs) [48]. Collectively, these findings suggest that the observed antibacterial activity of the fungal fermentation products may be attributable, at least in part, to the production or modulation of oxymatrine or structurally/functionally related secondary metabolites.

Furthermore, the flavonoids detected in the fungal fermentation product, including kaempferol, sakuranetin, and hesperetin, have also been reported in the plant *Euchresta tubulosa*. These compounds hold significant potential for medicinal research, as accumulating evidence suggests they possess various pharmacological activities, such as anti-inflammatory, antioxidant, neuroprotective, and cardiovascular disease-fighting effects. [49,50,51]. This study further evaluated the hydroxyl (•OH) and ABTS radical scavenging capacities of extracts derived from three endophytic fungal strains. Results revealed that all three strains exhibited concentration-dependent scavenging activity against both radicals; however, their •OH radical scavenging capacity was consistently lower than that of the positive control (ascorbic acid, Vc). Notably, the ethyl acetate extract obtained from the fermentation broth of ETY-2-B-b-II.1, an endophytic fungus isolated from the leaves of *E. tubulosa*, demonstrated significantly stronger ABTS radical scavenging activity than the Vc control at concentrations exceeding 1 mg/mL. Taken together with previous findings showing that the total flavonoid extract from *E. tubulosa* leaves exhibits markedly superior ABTS radical scavenging activity relative to Vc, these results suggest that the antioxidant properties of fermentation products derived from *E. tubulosa* endophytes may be attributable, at least in part, to flavonoid compounds present in their secondary metabolites [18].

These findings suggest that the endophytic fungi of *Euchresta tubulosa* could serve as a sustainable alternative resource to alleviate the shortage of plant resources. The bioactive metabolites produced by these fungi exhibit significant potential for development and promising application prospects in the research and development of novel pharmaceuticals, as well as in the investigation of their biological activities.

Extracellular enzymes, since their discovery, have been extensively studied for their roles in pathogenicity and substrate degradation. Notably, microbial production of proteases, amylases, carboxymethyl cellulases has been widely applied in fields such as edible mushroom cultivation, baking, food processing, biofuels, and healthcare [17,22,52,53]. As a result, they have gradually become the primary source of industrial enzyme preparations. Referring to the method of Shubha Jagannath et al. [37], specific criteria for the enzymatic zone of activity (ZA) of endophytic fungi were established. Notably, ZA less than 0.69 was defined as indicative of strong enzyme-producing capability. Our extracellular enzyme activity assays revealed that three *Euchresta tubulosa* endophytic strains possess a rich repertoire of extracellular enzymes.

The strains ETG-1-2-1 and ETXG-1-1-1 both exhibit high protease production capabilities. After 2 days of cultivation, their ZA consistently remained below 0.69, with the optimal ZA as low as 0.48, indicating that their protease production capacity at peak performance surpasses that of many other strains, including *Diaporthe* sp. KaL-5 [54], 26 strains of *Dendrobium officinale* seed endophytic fungi [55], and 54 strains of *Baliospermum montanum* endophytic fungi [37]. Furthermore, referencing the enzyme index (EI) evaluation criteria used by Chien Hao Chai et al. [56] or Tang [55], the assertion that these two fungal strains are strong protease producers is further validated.

Strain ETG-1-2-1 exhibited strong amylase production capacity during the early cultivation stage, by ZA consistently remaining below 0.69 within the first three days. Previous studies have reported that *Crinipellis perniciosa*, another member of the *Marasmiaceae* family, also demonstrated varying degrees of amylase production in solid media [57]. However, *Marasmius* sp. CE25, a strain known for its exceptionally high laccase activity, tested negative for amylase activity [58]. Furthermore, few strains within the *Marasmiaceae* family have been documented to possess high amylase production capabilities, and no studies have yet been conducted to optimize their enzyme production conditions. Given the strong amylase production capacity demonstrated by strain ETG-1-2-1 in this study, it is hypothesized that further optimization of enzyme production conditions could establish it as a model strain for high-yield amylase production within the *Marasmiaceae* family.

Three endophytic fungal from *Euchresta tubulosa* demonstrated a strong capacity for producing carboxymethyl cellulase (CMCase). Notably, strain ETXG-1-1-1 maintained exceptionally high CMCase production throughout its entire growth cycle, with the ZA consistently below 0.45. This performance surpassed that of three other *Talaromyces* fungi strains isolated from maize in terms of CMCase production capability [59]. Research by Shivam Aggarwal et al. [60] indicates that *Talaromyces* sp. is a novel fungus characterized by highly efficient cellulose degradation, with cellulases present on its spore surface. Their study showed that adding an inducer with a cellobiose-to-gentiobiose stoichiometric ratio of 2.5:1 to the culture medium could enhance the CMCase synthesis capability of *Talaromyces* sp. by more than 1.6-fold. This finding further supports the view that cellulases are adaptive enzymes whose expression is influenced by soluble inducers. Furthermore, studies have suggested that enzyme preparations from different fungi are superior to enzyme mixtures from a single strain [61]. Therefore, to obtain an optimal cellulose-degrading enzyme system, it is necessary to further explore and screen for CMCase-producing strains from *E*. *tubulosa*.

Endophytic fungi possess significant potential for producing industrially valuable enzymes. Based on our findings, the endophytic fungi isolated from *Euchresta tubulosa* exhibit considerable diversity in hydrolytic enzymes. Further quantitative analysis via submerged fermentation (SmF) demonstrated significant variations in the activities of protease, amylase, and carboxymethyl cellulase (CMCase) produced by three strains of *E*. *tubulosa* endophytic fungi.

The strains exhibited comparable protease-producing activities, with peak protease activities of ETG-1-2-1, ETY-2-B-b-II.1, and ETXG-1-1-1 being 1.26 ± 0.08 U/mL, 1.04 ± 0.06 U/mL, and 2.34 ± 0.14 U/mL, respectively. The differences in protease activity among the three strains are similar to those observed during solid-state fermentation (SSF). However, in comparison with studies by Camila Agnes Lumi Abe [59], Zhang [62], Cai [63], et al., the protease activities produced by these three endophytic fungi are considered to be at a moderate activity level. Further optimization of culture conditions for enzyme production is still required. Additionally, this further validates that even among same microbial origin different strains may exhibit functional divergence in nitrogen utilization and protein degradation [64].

Strain ETG-1-2-1 demonstrates exceptional polysaccharide-degrading capacity, with both its amylase and carboxymethyl cellulase (CMCase) activities being significantly higher than those of the other two strains. Amylase activity peaked at 10.95 ± 0.38 U/mL on day 4, while CMCase activity reached its maximum of 9.68 ± 0.305 U/mL on day 3. Compared with studies by Shivam Aggarwal [60], Honghai Zhang [65], and Shan Zhang [62], the amylase and CMCase activities of this strain have reached a strong activity level. In contrast, strain ETY-2-B-b-II.1 exhibited amylase and CMCase activities of 6.89 ± 0.66 U/mL and 6.17 ± 0.15 U/mL, respectively, with its amylase activity classified as strong, meanwhile its CMCase activity remained at a moderate level. The difference in polysaccharide degradation capabilities between the two strains likely reflects their occupancy of distinct ecological niches within the host microenvironment.

Notably, the amylase produced by strain ETXG-1-1-1 exhibited high activity during submerged fermentation (SmF), whereas the strain not only grew slowly but also showed negligible amylase activity in solid-state fermentation (SSF). For carboxymethyl cellulase (CMCase), the variation in ZA in solid culture indicated that this strain had an extremely high enzyme-producing potential; however, its activity only peaked at 3.00 ± 0.06 U/mL on the 4 d of liquid culture, representing the lowest activity among the three fungal strains tested. Given that the medium components were identical across all experiments, it is inferred that such differences in enzyme activity were caused by the variations in culture methods. This is consistent with the findings of numerous previous studies. For instance, Duan et al. [66] SSF and liquid—state fermentation (LSF) for cellulase production by *Trichoderma viride*, and the results showed that the yield of SSF was 25% higher than that of SmF, with a correspondingly higher enzyme output. In another study conducted by Cai et al. [63], the protease activity produced by *Rhizopus chinensis* under SSF was 9.2 times higher than that under SmF. Additionally, Hiroyuki Imanaka et al. [67] demonstrated that agar-plate cultivation or near-solid-state membrane-surface liquid cultivation could significantly stimulate *Aspergillus oryzae* to produce higher levels of protease and amylase. Furthermore, Cai et al. [63] employed two-dimensional gel electrophoresis combined with mass spectrometry to separate and identify extracellular proteins produced by *Rhizopus chinensis* under solid-state and submerged cultivation. The results demonstrated that the extracellular proteins generated by *Rhizopus chinensis* under the two cultivation systems exhibited significant differences in both types and expression levels, with approximately 70% being specific proteins produced under either solid-state or submerged cultivation, among which hydrolases accounted for a relatively large proportion. In addition, building on the findings of Hiroyuki Imanaka et al. [67], which demonstrated that most genes specifically transcribed under membrane-surface liquid cultivation were upregulated by 10-fold or more compared with those specifically transcribed under shake-flask cultivation—it can be concluded that the differences in enzyme activity detected in strain ETXG-1-1-1 under solid-state and submerged cultivation are mainly attributed to the distinct enzyme-producing mechanisms of the strain under different culture methods.

The results of this study further confirm the potential of endophytic fungi in the production of industrial enzymes. Further in-depth analysis of the regulatory mechanisms governing extracellular enzyme activity in endophytic fungi and scientific optimization of the optimal enzyme-producing conditions for strains are required, so as to lay a solid scientific foundation for the application of endophytic fungi in the food processing industry, pharmaceutical manufacturing, and various biomedical therapies.

## 5. Conclusions

This study presents a systematic investigation of endophytic fungi isolated from *Euchresta tubulosa* Dunn, an endangered ethnomedicinal plant, and encompassing their isolation, taxonomic identification, secondary metabolites profiling, extracellular enzyme activities assays. Using the tissue explant culture approach, 55 endophytic fungal strains were obtained and classified into 17 genera, based on morphological and molecular characteristics, with *Colletotrichum*, *Fusarium*, and *Penicillium* representing the dominant genera. Among these, four strains were proposed as putative new species, including three novel taxa. Metabolite analysis revealed of fermentation broths from three potentially novel species (or genera) revealed abundant medically relevant bioactive compounds, such as flavonoids and alkaloids. Combined with bioactivity assays, the fermentation products of the three bacterial strains exhibited varying degrees of antibacterial and antioxidant activity. Analysis revealed that the secondary metabolites of these strains are closely associated with their biological activities. Extracellular enzyme activity assays indicated that some strains exhibited broad-spectrum substrate hydrolysis capabilities. Notably, strain ETG-1-2-1 exhibited outstanding polysaccharide-degrading capacity, demonstrating the highest amylase and cellulase activities (10.95 U/mL and 9.68 U/mL, respectively), while strain ETXG-1-1-1 demonstrated prominent protease activity (2.34 U/mL) and maintained a strong ability to produce carboxymethyl cellulase (CMCase) over prolonged periods under solid-state fermentation (SSF) conditions. This study provides the first comprehensive report on the diversity, metabolic characteristics, and ecological functions of endophytic fungi in *E*. *tubulosa*. These findings establish a foundation for the future exploration of bioactive natural products, development of enzymatic preparations, and utilization of microbial resources derived from endophytic fungi.

## Figures and Tables

**Table 1:** Results of sequence comparison of ITS segments of three endophytic fungi from the *Euchresta tubulosa*.

Number of Fungi	Abbreviation	Site	GenBank ID	Scientific Name	Accession Number
ETG-1-2-1	ETG	Root	PP702993	*Neocrinipellis albifloccum*	CGMCC NO.41962
ETY-2-B-b-II.1	ETY	Leaf	PP669799	*Xylaria cinereonitens*	CGMCC NO. 42144
ETXG-1-1-1	ETXG	Fibrous root	PP702998	*Talaromyces polymorphus*	CGMCC NO. 41927

**Table 2:** Colonial morphological characteristics of three endophytic fungi isolated from *Euchresta tubulosa*.

Number of Fungi	Isolation Source	Colony Color	Margin Color	Texture	Growth Rate Regression Equation	R^2^
ETG-1-2-1	Root	White	White	Floccose	y = 0.96051x + 0.37713	0.9956
ETY-2-B-b-II.1	Leaf	Grayish-black	Gray	Velvety	y = 0.76386x + 0.13007	0.9997
ETXG-1-1-1	Fibrous root	Red	White	Floccose	y = 0.55317x + 0.12988	0.9994

**Table 3:** Selected metabolites detected by LC–MS/MS in endophytic fungi.

Class	Compounds	Formula	Molecular Weight	RT (min)	*m*/*z*	Source
Alkaloids	Reserpine	C_33 _H_40_N_2_O_9_	608.27772	3.813	607.27045	ETG/ETY/ETXG
	Guvacoline	C_7_H_11_NO_2_	141.07930	4.656	142.08658	ETG/ETY/ETXG
	Pilocarpine	C_11_H_16_N_2_O_2_	208.12120	3.047	226.15500	ETG/ETY/ETXG
	Piperine	C_17_H_19_NO_3_	285.13262	5.242	286.14362	ETG/ETY/ETXG
	Trigonelline	C_7_H7NO2	137.04769	1.397	138.05496	ETXG/ETG
	Oxymatrine	C_15_H_24_N_2_O_2_	264.18389	4.974	265.19116	ETXG/ETG
	Gelsemine	C_20_H_22_N_2_O_2_	322.16399	5.320	323.17126	ETXG/ETG
Flavonoids	Glycitein	C_16_H_12_O_5_	284.06828	5.958	285.07556	ETG/ETY/ETXG
	Daidzein	C_15_H_10_O_4_	254.05794	5.951	255.06522	ETXG/ETG
	Kaempferol	C_15_H_10_O_6_	286.04793	6.184	285.04065	ETY/ETXG
	4′,7-Dihydroxyflavanone	C_15_H_12_O_4_	256.07350	5.848	257.08078	ETY
	Sakuranetin	C_16_H_14_O_5_	286.08422	5.866	287.09149	ETY
	Genistein	C_15_H_10_O_5_	270.05354	6.10 7	269.04626	ETXG
	Rotenone	C_23_H_22_O_6_	394.14189	8.458	393.13461	ETXG
	Isorhamnetin	C_16_H_12_O_7_	316.06129	6.592	315.05402	ETG
	3-Methoxyflavone	C_16_H_12_O_3_	274.06240	1.853	292.09607	ETG
	Hesperetin	C_16_H_14_O_6_	302.07915	5.575	301.07187	ETY/ETG
	Naringenin	C_15_H_12_O_5_	272.06854	5.760	253.05077	ETG
	Formononetin	C_16_H_12_O_4_	268.07347	5.982	269.08075	ETY/ETG
	Troxerutin	C_33_H_42_O_19_	780.18053	11.064	781.18781	ETG
Tetracyclines	Chlortetracycline	C_22_H_23_ClN_2_O_8_	478.11369	6.173	477.10641	ETG
	Tetracycline	C_22_H_24_N_2_O_8_	444.14949	2.794	443.14221	ETY
	Minocycline	C_23_H_27_N_3_O_7_	457.18138	2.713	458.18866	ETG/ETY
Stilbenes	Isorhapontigenin	C_15_H_14_O_4_	258.08516	4.011	257.07788	ETG/ETY/ETXG
Terpenoids	(-)-Caryophyllene oxide	C_15_H_24_O	220.18297	6.562	221.19025	ETG/ETY/ETXG
	Ethyl chrysanthemumate	C_12_H_20_O_2_	196.14577	6.043	197.15305	ETG/ETY/ETXG
	Perillic acid	C_10_H_14_O_2_	166.09952	5.359	165.09224	ETG/ETXG
	Gibberellin A7	C_19_H_22_O_5_	330.14299	5.635	329.13571	ETXG/ETY
	Carvone	C_10_H_14_O	150.10454	6.823	151.11182	ETG
	Cryptotanshinone	C_19_H_20_O_3_	296.13713	5.743	279.13388	ETXG
	Gibberellin A4	C_19_H_24_O_5_	332.15877	4.676	331.15149	ETY
Steroids	Corticosterone	C_21_H_30_O_4_	346.21424	7.162	347.22174	ETG/ETY/ETXG
	11β-Hydroxyandrosterone	C_19_H_30_O_3_	306.21941	6.869	307.22672	ETG/ETY
	Desoxycortone	C_21_H_30_O_3_	330.21710	7.077	331.22437	ETG/ETXG
	Hydrocortisone	C_21_H_30_O_5_	362.20933	6.924	363.21631	ETXG/ETY
	Diflorasone	C_22_H_28_F_2_O_5_	410.18997	2.524	391.17197	ETY

**Table 4:** Minimum inhibitory concentration (MIC) of fermentation products from endophytic fungi of *Euchresta tubulosa* on three indicator strains.

Number	MIC (mg/mL)		
	*Escherichia coli*	*Staphylococcus aureus*	*Candida albicans*
ETG-1-2-1	2.5	2.5	1.25
ETXG-1-1-1	5	2.5	2.5
ETY-2-B-b-II.1	5	2.5	5

## Data Availability

The raw data supporting the conclusions of this article will be made available by the authors on request.

## Supplementary Materials

The following supporting information can be downloaded at: https://www.mdpi.com/article/10.3390/microorganisms14030664/s1, Figure S1: TIC profiles of sample ETG-1-2-1. (Top) Positive ESI mode (targeting cations); (bottom) negative ESI mode (targeting anions). Figure S2: TIC profiles of sample ETY-2-B-b-II.1. (Top) Positive ESI mode (targeting cations); (bottom) nnegative ESI mode (targeting anions). Figure S3: TIC profiles of sample ETXG-1-1-1. (Top) Positive ESI mode (targeting cations). Figure S4: Mass spectrum of pilocarpine. (TOP) Full scan mass spectrum of pilocarpine; (Bottom) MS/MS spectrum of pilocarpine. Figure S5: Mass spectrum of trigonelline. (Top) Full scan mass spectrum of trigonelline; (bottom) MS/MS spectrum of trigonelline. Figure S6: Mass spectrum of oxymatrine. (Top) Full scan mass spectrum of oxymatrine; (bottom) MS/MS spectrum of oxymatrine. Figure S7: Mass spectrum of glycitein. (Top) Full scan mass spectrum of glycitein; (bottom) MS/MS spectrum of glycitein. Figure S8: Mass spectrum of 4′,7-Dihydroxyflavanone. (Top) Full scan mass spectrum of 4′,7-Dihydroxyflavanone; (bottom) MS/MS spectrum of 4′,7-Dihydroxyflavanone. Figure S9: Mass spectrum of naringenin. (Top) Full scan mass spectrum of naringenin; (bottom) MS/MS spectrum of naringenin. Figure S10: Mass spectrum of formononetin. (Top) Full scan mass spectrum of formononetin; (bottom) MS/MS spectrum of formononetin. Figure S11: Mass spectrum of minocycline. (Top) Full scan mass spectrum of minocycline; (bottom) MS/MS spectrum of minocycline. Figure S12. Results of sequence comparison of ITS segments of endophytic fungi from ETG-1-2-1. Figure S13. Results of sequence comparison of ITS segments of endophytic fungi from ETY-2-B-b-II.1. Figure S14. Results of sequence comparison of ITS segments of endophytic fungi from ETXG-1-1-1. Figure S15. Results of sequence comparison of ITS segments of endophytic fungi from ETXG-1-3-1. Figure S16. Results of ITS sequence alignment from ETXG-1-1-1 and ETXG-1-3-1. Figure S17. Standard curve of determination of content glucose (A) and tyrosine (B). Table S1. Preparation of Medium.

## Abbreviations

The following abbreviations are used in this manuscript:

*E. tubulosa*	*Euchresta tubulosa* Dunn
LC–MS/MS	Liquid Chromatography–Tandem Mass Spectrometry
PDA	Potato Dextrose Agar
PDB	Potato Dextrose Broth
ZA	Enzymatic zone of activity
ETG	ETG-1-2-1
ETY	ETY-2-B-b-II.1
ETXG	ETXG-1-1-1
CMC-Na	Carboxymethyl cellulose sodium
CMCase	Carboxymethyl cellulase
SSF	Solid-state fermentation
SmF	Submerged fermentation
