# Supplementary material for: Novel Endophytic Fungi from Euchresta tubulosa Dunn: Characterization of Their Bioactive Secondary Metabolites and Extracellular Enzymes
Source: Microorganisms. 2026 Mar 15;14(3):664. doi: 10.3390/microorganisms14030664 (PMC13029386; doi:10.3390/microorganisms14030664)
Supplement: Supplementary file 1 [file microorganisms-14-00664-s001.zip › microorganisms-4165123-supplementary.pdf]

SUPPLEMENTARY MATERIAL

Novel Endophytic Fungi from *Euchresta tubulosa* Dunn: Their Bioactive Secondary Metabolites and Extracellular Enzymes of Characterization

Xinlian Yin [1†], Wei Guo [1†], Qing Wang [1], Rushuang Nie[1], Dujiang Qing [1], Yao Hu [1], Sisi Hu [1], Linxin Wang [1], Xiaolin Ye [1], Shufeng Yao [1, 2*], Jiang Cheng [1, 2*]

[1] School of Pharmaceutical Sciences, Jishou University, Jishou 416000, Hunan, China

[2] Key Laboratory of Medicinal Resources Chemistry and Pharmacology in Wuling Mountainous of Hunan Province College, Jishou University, Jishou 416000, Hunan, China

[*]Corresponding author: ysf2022@jsu.edu.cn(S.F. Yao); awb_cj@jsu.edu.cn(J. Cheng)

[†]These authors contributed equally to this work.

Table of contents

Figure S1. TIC profiles of sample ETG-1-2-1 . (Top) Positive ESI mode (targeting cations) ; (Bottom) Negative ESI mode (targeting anions). 3
Figure S2. TIC profiles of sample ETY-2-B-b-II.1 . (Top) Positive ESI mode (targeting cations) ; (Bottom) Negative ESI mode (targeting anions). 3
Figure S3. TIC profiles of sample ETXG-1-1-1 . (Top) Positive ESI mode (targeting cations) ;...4
Figure S4. Mass spectrum of Pilocarpine. (TOP)Full scan mass spectrum of Pilocarpine; (Bottom)MS/MS spectrum of Pilocarpine...4
Figure S5. Mass spectrum of Trigonelline . (TOP)Full scan mass spectrum of Trigonelline ; (Bottom)MS/MS spectrum of Trigonelline...5
Figure S6. Mass spectrum of Oxymatrine . (TOP)Full scan mass spectrum of Oxymatrine ; (Bottom)MS/MS spectrum of Oxymatrine..5
Figure S7. Mass spectrum of the Glycitein . (TOP)Full scan mass spectrum of Glycitein ; (Bottom)MS/MS spectrum of Glycitein 6
Figure S8. Mass spectrum of the 4',7-Dihydroxyflavanone . (TOP)Full scan mass spectrum of 4',7-Dihydroxyflavanone ; (Bottom)MS/MS spectrum of 4',7-Dihydroxyflavanone 6
Figure S9. Mass spectrum of the Naringenin . (TOP)Full scan mass spectrum of Naringenin ; (Bottom)MS/MS spectrum of Naringenin 7
Figure S10. Mass spectrum of the Formononetin . (TOP)Full scan mass spectrum of Formononetin ; (Bottom)MS/MS spectrum of Formononetin.....................................7
Figure S11. Mass spectrum of the Minocycline . (TOP)Full scan mass spectrum of Minocycline ; (Bottom)MS/MS spectrum of Minocycline..8
Figure S12. Results of sequence comparison of ITS segments of endophytic fungi from ETG-1-2-1 8
Figure S13. Results of sequence comparison of ITS segments of endophytic fungi from ETY-2-B-b-II.1. 9
Figure S14. Results of sequence comparison of ITS segments of endophytic fungi from ETXG-1-1-1. 9
Figure S15. Results of sequence comparison of ITS segments of endophytic fungi from ETXG-1-3-1 10
Figure S16. Results of ITS sequence alignment from ETXG-1-1-1 and ETXG-1-3-1 10
Table S1. Preparation of Medium...11
Figure S17. Standard curve of determination of content glucose(A) and tyrosine (B). 13

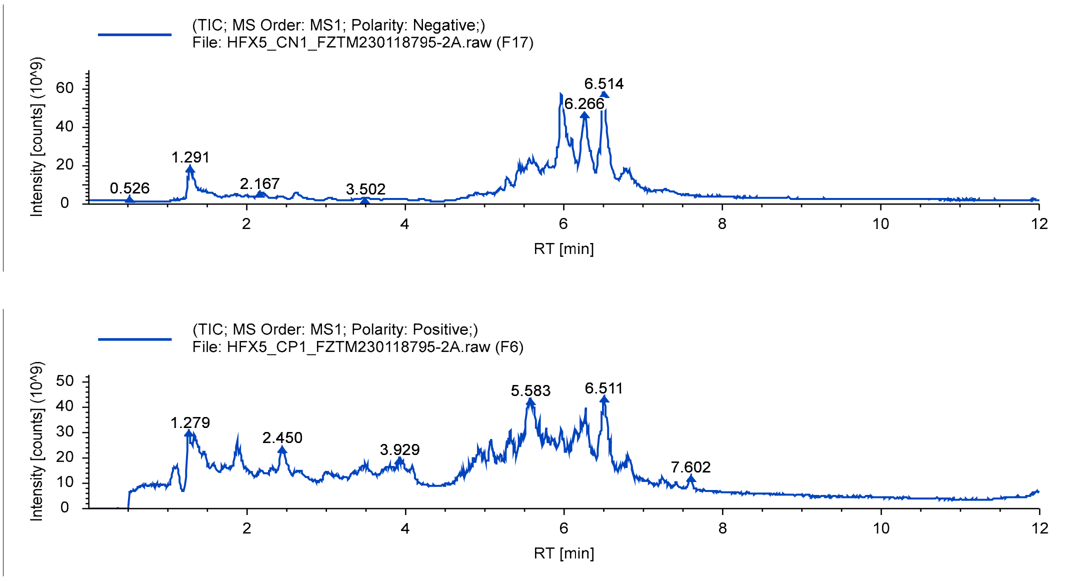

Figure S1. TIC profiles of sample ETG-1-2-1 . (Top) Positive ESI mode (targeting cations) ; (Bottom) Negative ESI mode (targeting anions).

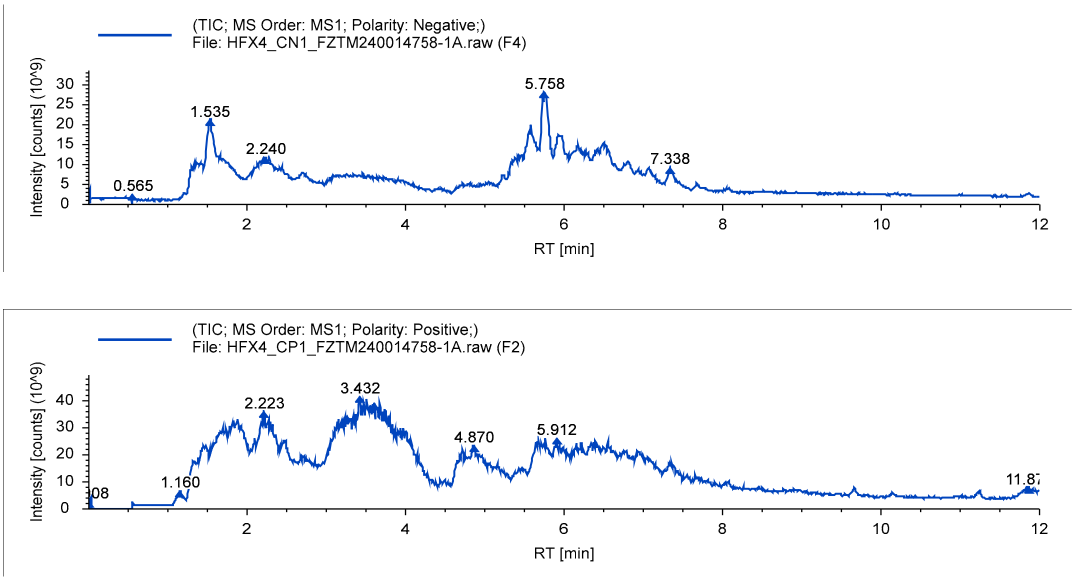

Figure S2. TIC profiles of sample ETY-2-B-b-II.1 . (Top) Positive ESI mode (targeting cations) ; (Bottom) Negative ESI mode (targeting anions).

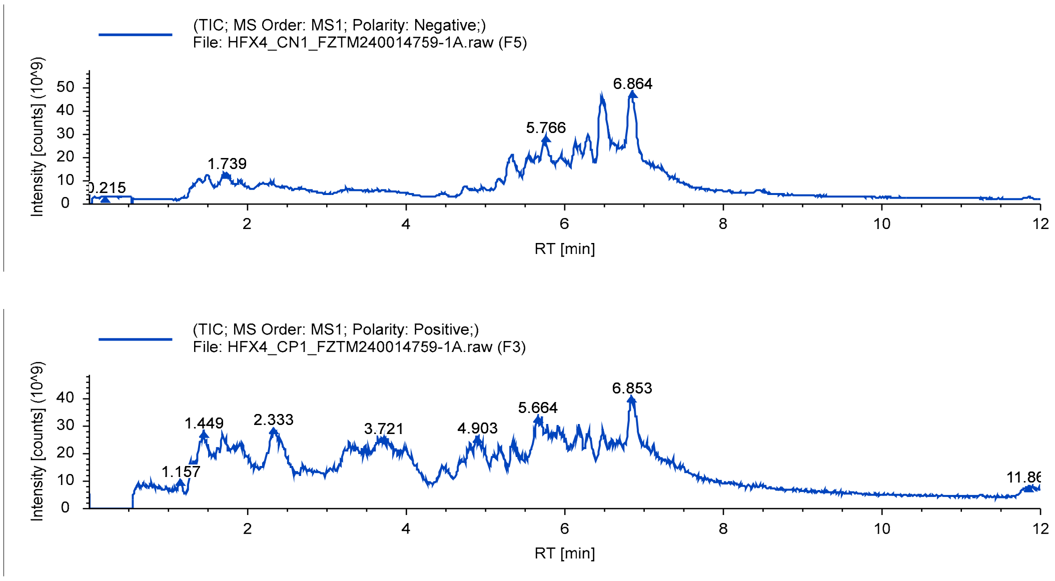

Figure S3. TIC profiles of sample ETXG-1-1-1 . (Top) Positive ESI mode (targeting cations) ; (Bottom) Negative ESI mode (targeting anions).

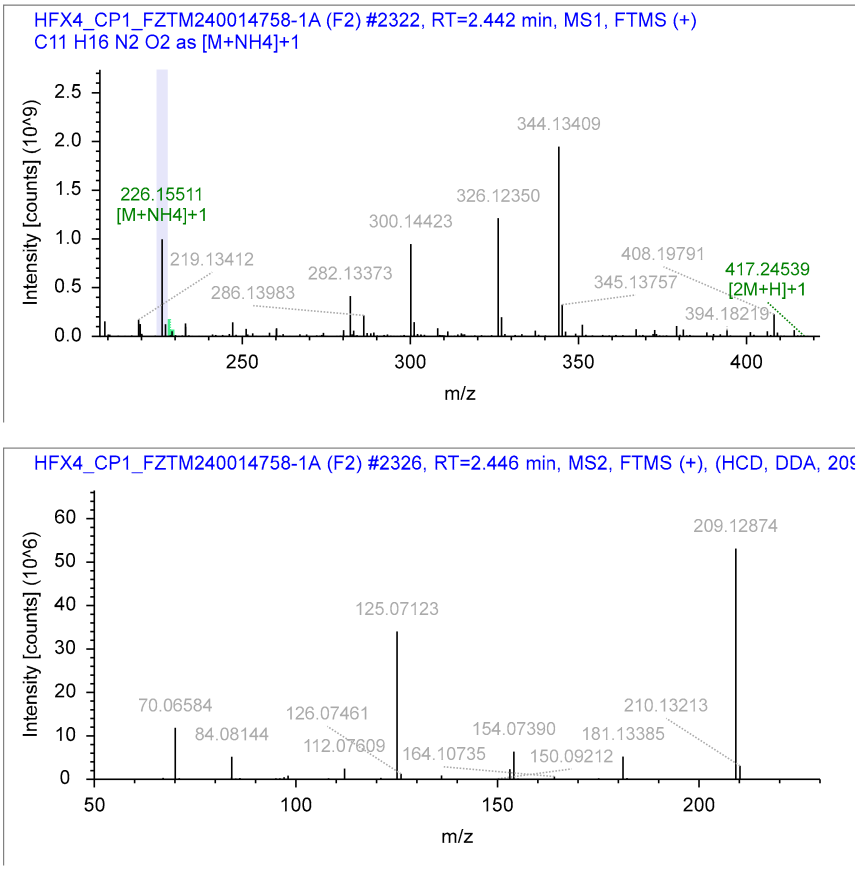

Figure S4. Mass spectrum of Pilocarpine. (TOP)Full scan mass spectrum of Pilocarpine; (Bottom)MS/MS spectrum of Pilocarpine.

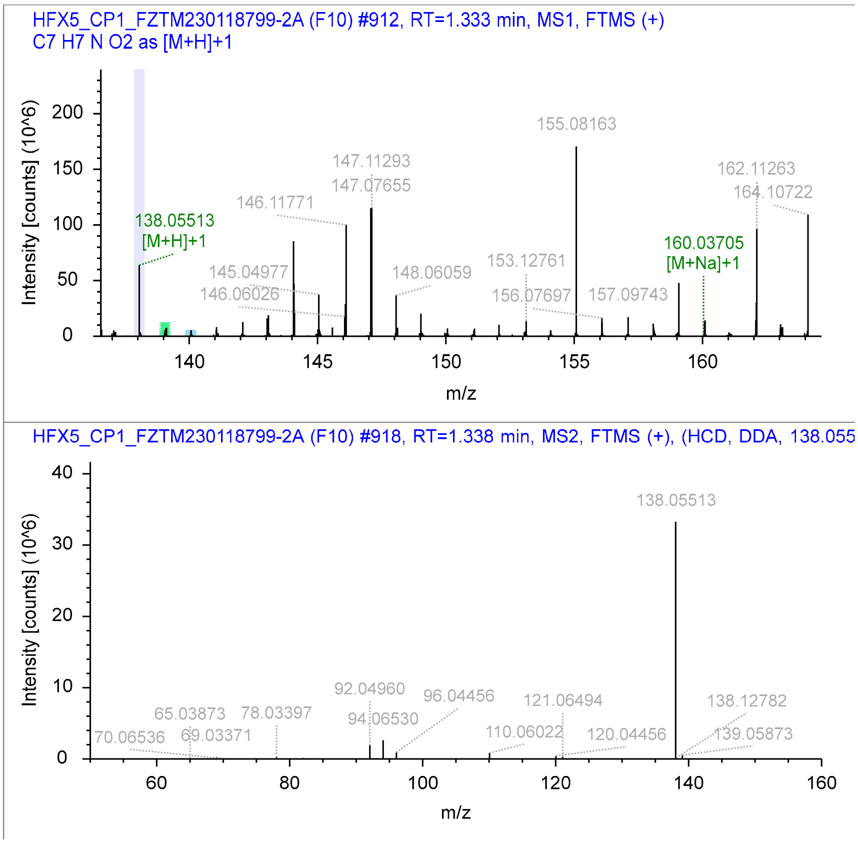

Figure S5. Mass spectrum of Trigonelline . (TOP)Full scan mass spectrum of Trigonelline ; (Bottom)MS/MS spectrum of Trigonelline.

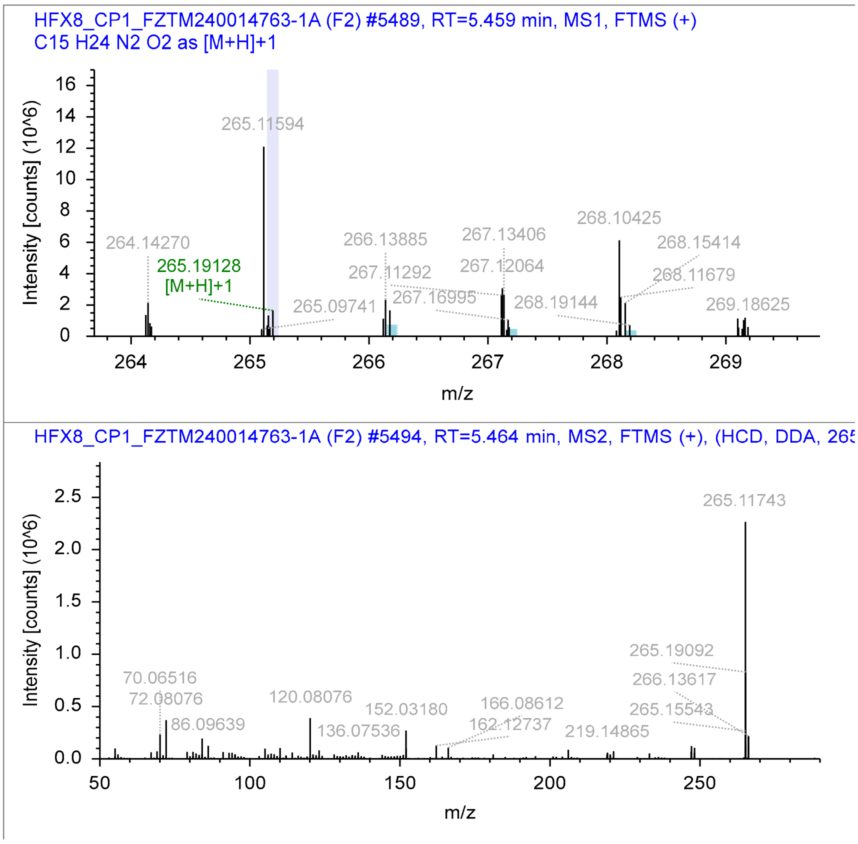

Figure S6. Mass spectrum of Oxymatrine . (TOP)Full scan mass spectrum of Oxymatrine ; (Bottom)MS/MS spectrum of Oxymatrine.

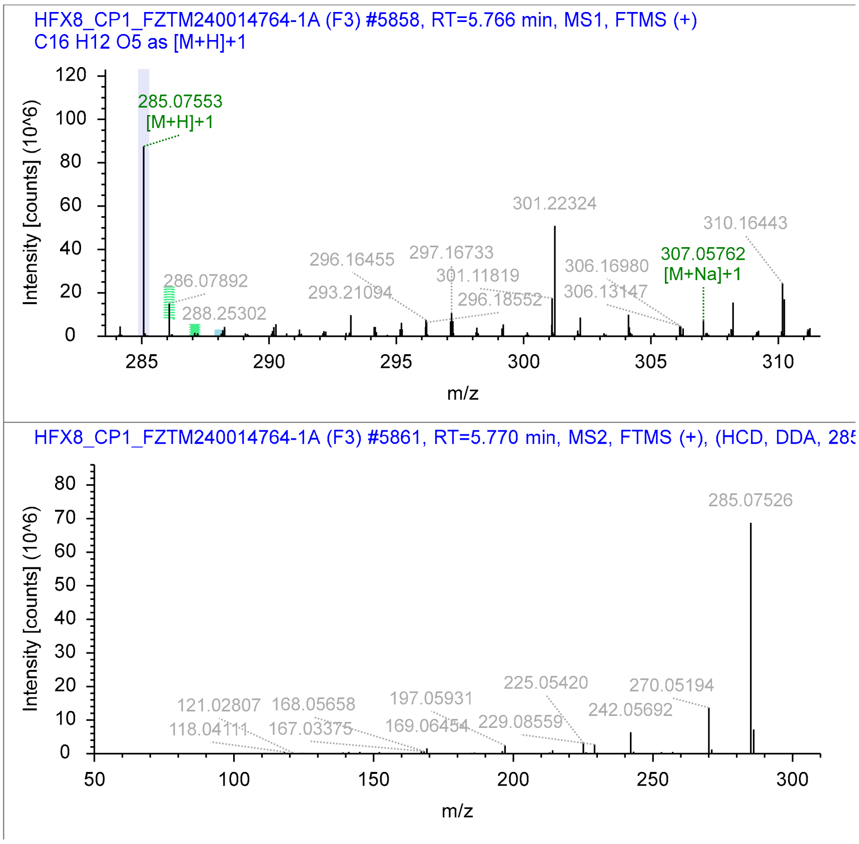

Figure S7. Mass spectrum of the Glycitein . (TOP)Full scan mass spectrum of Glycitein ; (Bottom)MS/MS spectrum of Glycitein

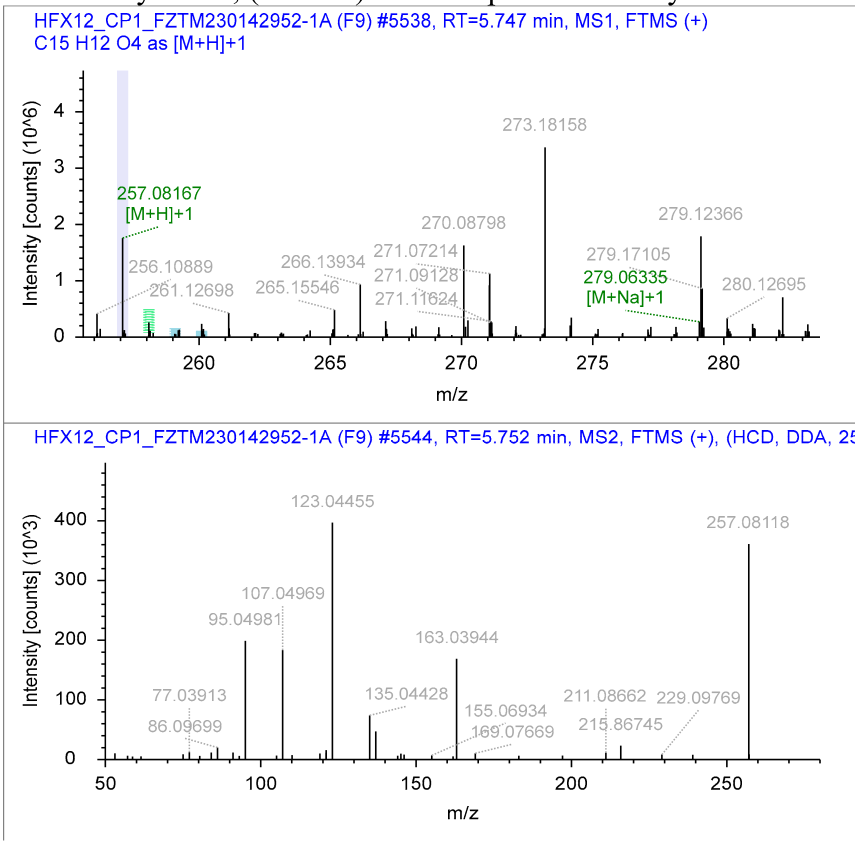

Figure S8. Mass spectrum of the 4',7-Dihydroxyflavanone . (TOP)Full scan mass spectrum of 4',7-Dihydroxyflavanone ; (Bottom)MS/MS spectrum of 4',7-Dihydroxyflavanone

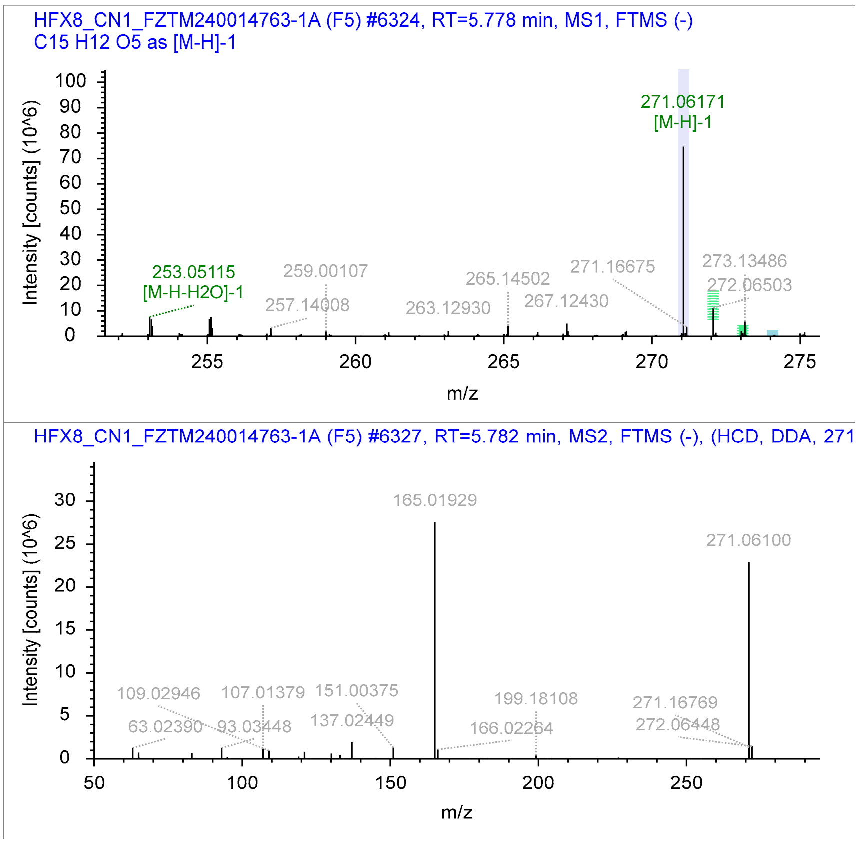

Figure S9. Mass spectrum of the Naringenin . (TOP)Full scan mass spectrum of Naringenin ; (Bottom)MS/MS spectrum of Naringenin

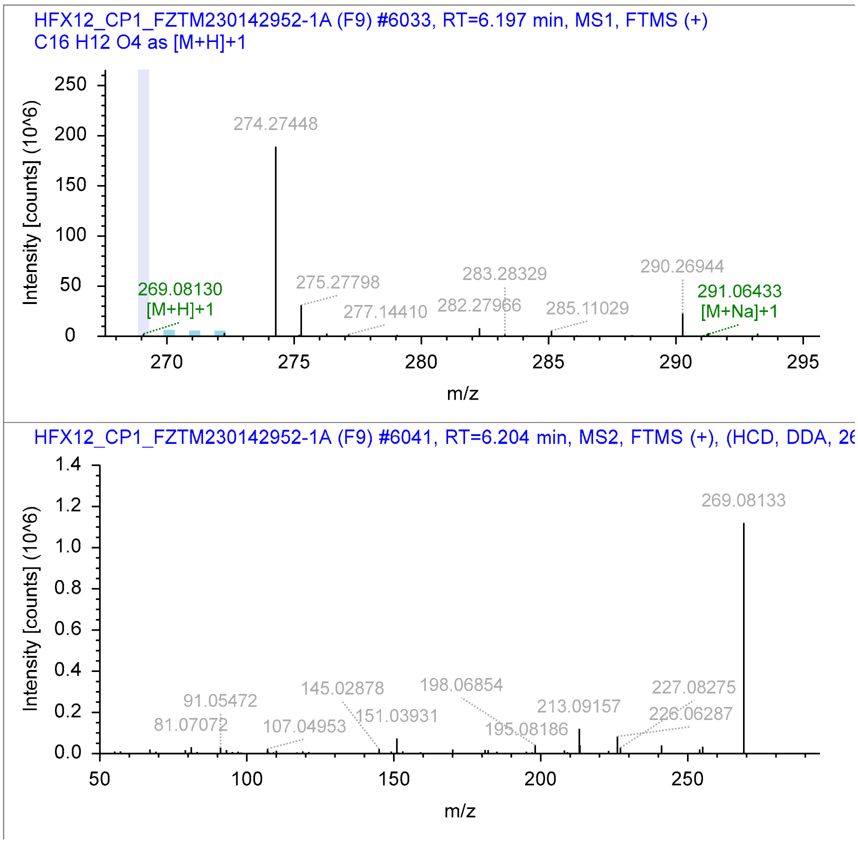

Figure S10. Mass spectrum of the Formononetin . (TOP)Full scan mass spectrum of Formononetin ; (Bottom)MS/MS spectrum of Formononetin.

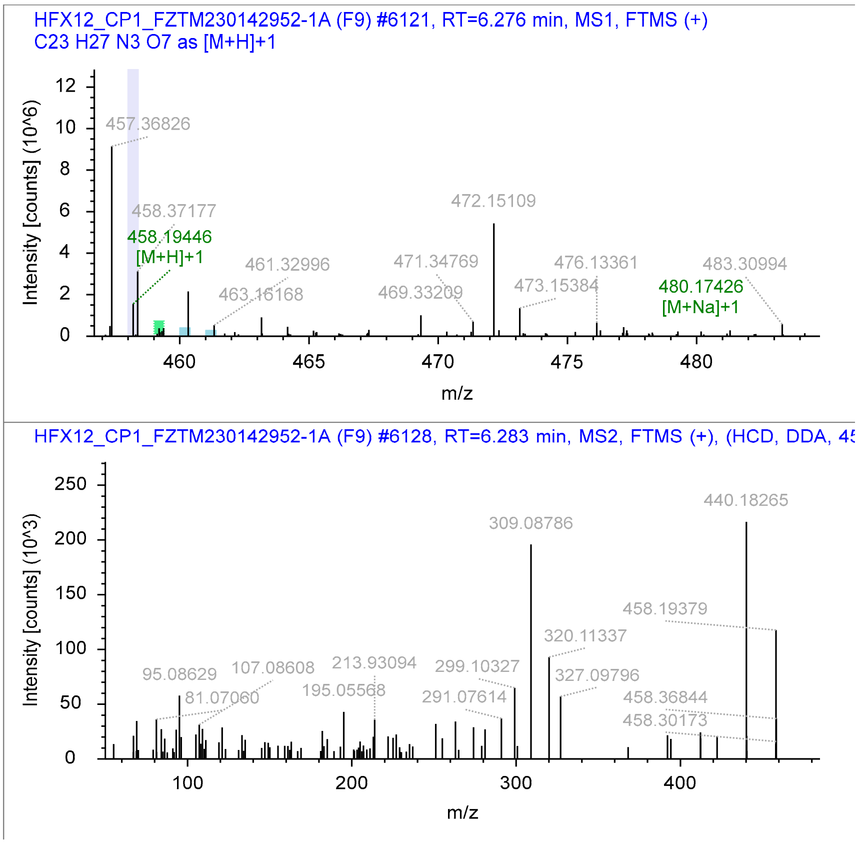

Figure S11. Mass spectrum of the Minocycline . (TOP)Full scan mass spectrum of Minocycline ; (Bottom)MS/MS spectrum of Minocycline

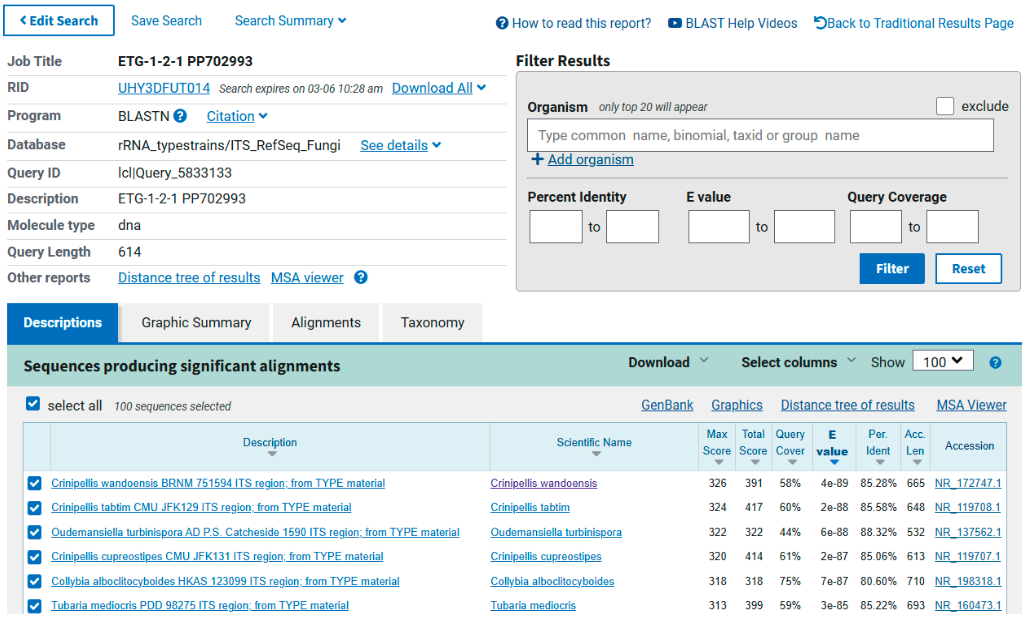

Description	Scientific Name	Max Score	Total Score	Query Cover	E value	Per. Ident	Acc. Len	Accession
Crinipellis wandoensis BRNM 751594 ITS region; from TYPE material	Crinipellis wandoensis	326	391	58%	4e-89	85.28%	665	NR_172747.1
Crinipellis tabtim CMU JFK129 ITS region; from TYPE material	Crinipellis tabtim	324	417	60%	2e-88	85.58%	648	NR_119708.1
Oudemansiella turbinispora AD P.S. Catcheside 1590 ITS region; from TYPE material	Oudemansiella turbinispora	322	322	44%	6e-88	88.32%	532	NR_137562.1
Crinipellis cupreostipes CMU JFK131 ITS region; from TYPE material	Crinipellis cupreostipes	320	414	61%	2e-87	85.06%	613	NR_119707.1
Collybia alboclitocyboides HKAS 123099 ITS region; from TYPE material	Collybia alboclitocyboides	318	318	75%	7e-87	80.60%	710	NR_198318.1
Tubaria mediocris PDD 98275 ITS region; from TYPE material	Tubaria mediocris	313	399	59%	3e-85	85.22%	693	NR_160473.1

Figure S12. Results of sequence comparison of ITS segments of endophytic fungi from ETG-1-2-1

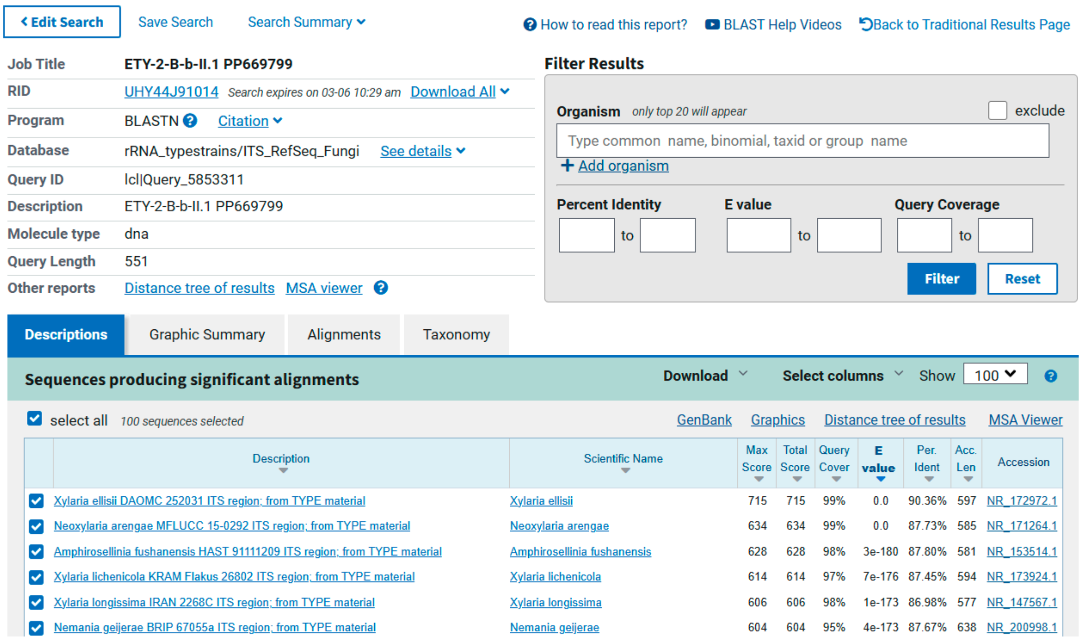

Edit Search | Save Search | Search Summary | How to read this report? | BLAST Help Videos | Back to Traditional Results Page

Job Title	ETY-2-B-b-II.1 PP669799
RID	UHY44J91014 Search expires on 03-06 10:29 am Download All
Program	BLASTN Citation
Database	rRNA_typestrains/ITS_RefSeq_Fungi See details
Query ID	lcl|Query_5853311
Description	ETY-2-B-b-II.1 PP669799
Molecule type	dna
Query Length	551
Other reports	Distance tree of results MSA viewer

Filter Results

Organism only top 20 will appear exclude

Type common name, binomial, taxid or group name

Add organism

Percent Identity to | E value to | Query Coverage to

Filter Reset

Descriptions | Graphic Summary | Alignments | Taxonomy

Sequences producing significant alignments | Download | Select columns | Show 100

select all 100 sequences selected | GenBank | Graphics | Distance tree of results | MSA Viewer

Description	Scientific Name	Max Score	Total Score	Query Cover	E value	Per. Ident	Acc. Len	Accession
Xylaria ellisii DAOMC 252021 ITS region; from TYPE material	Xylaria ellisii	715	715	99%	0.0	90.36%	597	NR_172972.1
Neoxylaria arengae MFLUCC 15-0292 ITS region; from TYPE material	Neoxylaria arengae	634	634	99%	0.0	87.73%	585	NR_171264.1
Amphirosellinia fushanensis HAST 91111209 ITS region; from TYPE material	Amphirosellinia fushanensis	628	628	98%	3e-180	87.80%	581	NR_153514.1
Xylaria lichenicola KRAM Flakus 26802 ITS region; from TYPE material	Xylaria lichenicola	614	614	97%	7e-176	87.45%	594	NR_173924.1
Xylaria longissima IRAN 2268C ITS region; from TYPE material	Xylaria longissima	606	606	98%	1e-173	86.98%	577	NR_147567.1
Nemania geijerae BRIP 67055a ITS region; from TYPE material	Nemania geijerae	604	604	95%	4e-173	87.67%	638	NR_200998.1

Figure S13. Results of sequence comparison of ITS segments of endophytic fungi from ETY-2-B-b-II.1.

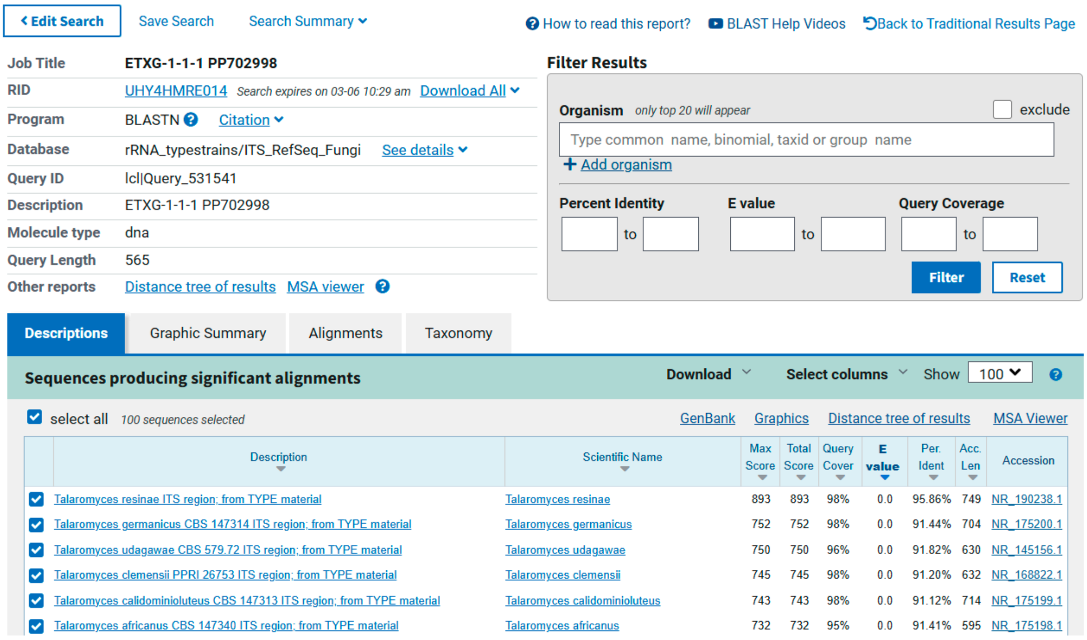

Edit Search | Save Search | Search Summary | How to read this report? | BLAST Help Videos | Back to Traditional Results Page

Job Title	ETXG-1-1-1 PP702998
RID	UHY4HMRE014 Search expires on 03-06 10:29 am Download All
Program	BLASTN Citation
Database	rRNA_typestrains/ITS_RefSeq_Fungi See details
Query ID	lcl|Query_531541
Description	ETXG-1-1-1 PP702998
Molecule type	dna
Query Length	565
Other reports	Distance tree of results MSA viewer

Filter Results

Organism only top 20 will appear exclude

Type common name, binomial, taxid or group name

Add organism

Percent Identity to | E value to | Query Coverage to

Filter Reset

Descriptions | Graphic Summary | Alignments | Taxonomy

Sequences producing significant alignments | Download | Select columns | Show 100

select all 100 sequences selected | GenBank | Graphics | Distance tree of results | MSA Viewer

Description	Scientific Name	Max Score	Total Score	Query Cover	E value	Per. Ident	Acc. Len	Accession
Talaromyces resinae ITS region; from TYPE material	Talaromyces resinae	893	893	98%	0.0	95.86%	749	NR_190238.1
Talaromyces germanicus CBS 147314 ITS region; from TYPE material	Talaromyces germanicus	752	752	98%	0.0	91.44%	704	NR_175200.1
Talaromyces udagawae CBS 579.72 ITS region; from TYPE material	Talaromyces udagawae	750	750	96%	0.0	91.82%	630	NR_145156.1
Talaromyces clemensii PPRI 26753 ITS region; from TYPE material	Talaromyces clemensii	745	745	98%	0.0	91.20%	632	NR_168822.1
Talaromyces calidominioluteus CBS 147313 ITS region; from TYPE material	Talaromyces calidominioluteus	743	743	98%	0.0	91.12%	714	NR_175199.1
Talaromyces africanus CBS 147340 ITS region; from TYPE material	Talaromyces africanus	732	732	95%	0.0	91.41%	595	NR_175198.1

Figure S14. Results of sequence comparison of ITS segments of endophytic fungi from ETXG-1-1-1.

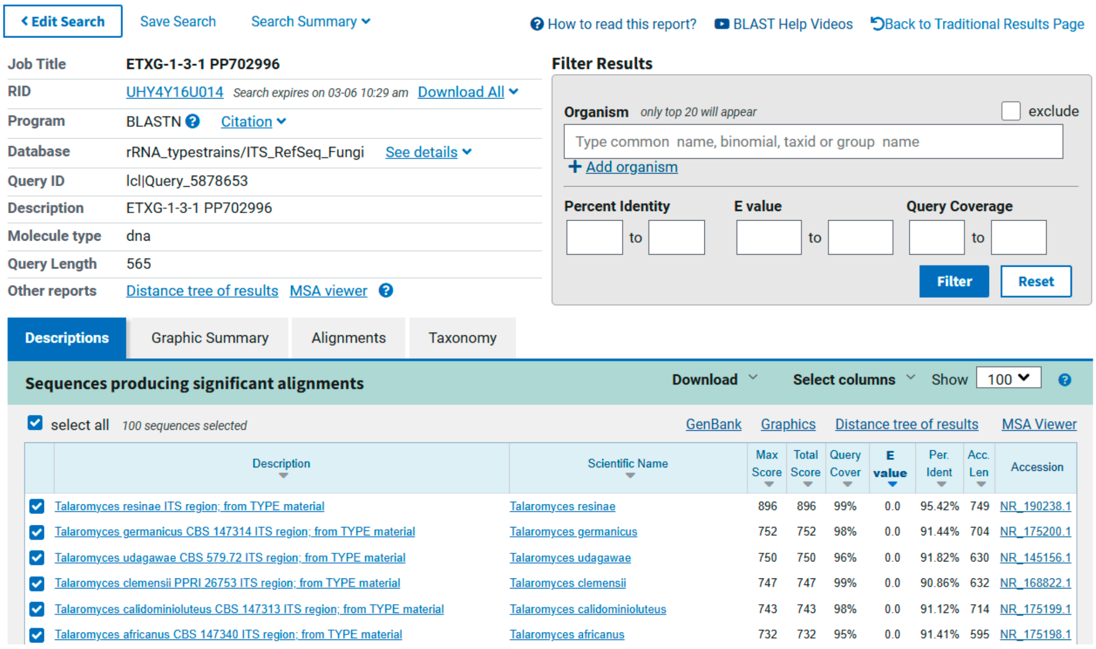

Figure S15. Results of sequence comparison of ITS segments of endophytic fungi from ETXG-1-3-1

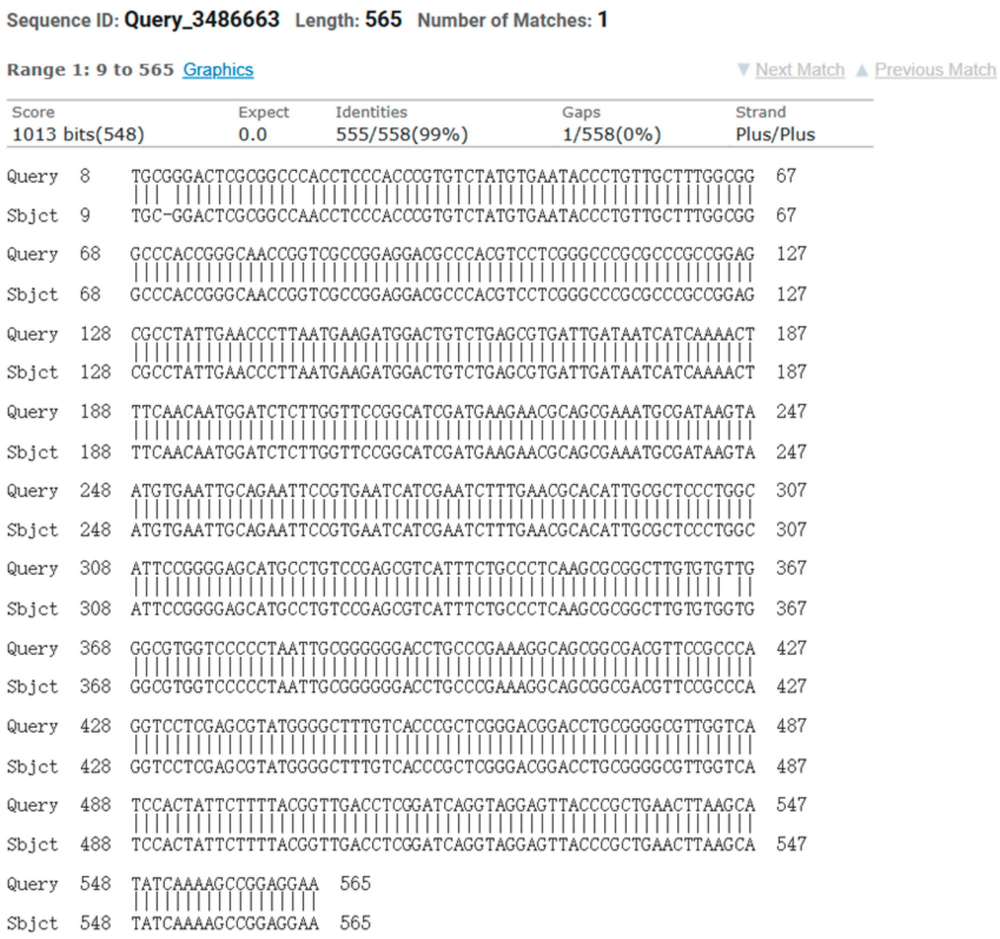

Figure S16. Results of ITS sequence alignment from ETXG-1-1-1 and ETXG-1-3-1

Table S1. Preparation of Medium

Types of culture media	Components of the medium	Preparation method
**PDA Medium**	Potato extract 6.0 g, Dextrose 20.0 g, Agar 20.0 g	Weigh 46.0 g of Potato Dextrose Agar (PDA) powder, dissolve in 1,000 mL distilled water, and autoclave at 121℃ for 15 min. Cool to 50 - 60 ℃, then add filter-sterilized (0.22 um) Penicillin G sodium salt and kanamycin solutions to achieve final concentrations of 150 mg/L each. Pour plates under aseptic conditions to solidify as solid media. For slants: After complete dissolution, dispense 1/3 volume into 18 mm test tubes with silicone stoppers. Autoclave at 121℃ for 15 min, then solidify at a 30℃ angle in a biosafety cabinet.
**Inorganic Salt Starch Medium**	solu-ble starch 10.0 g K_2HPO_4 1 g, $MgSO_4{\cdot}7H_2O$ 1 .0g, $(NH_4)_2SO_4$ 2 .0g, $CaCO_3$ 2 .0g, $FeSO_4$ 1 mg, $MnCl_2$ 1 mg, $ZnSO_4$ 1 mg, NaCl 1 g, and agar 20.0 g	Prepare starch solution: Gradually add 10 g solu-ble starch to boiling purified water with constant stirring until dissolved. Sepa-rately, dissolve K_2HPO_4 (1.0 g), $MgSO_4{\cdot}7H_2O$ (1.0 g), $(NH_4)_2SO_4$ (2.0 g), $CaCO_3$ (2.0 g), $FeSO_4$ (1 mg), $MnCl_2$ (1 mg), $ZnSO_4$ (1 mg), NaCl (1.0 g), and agar (20.0 g) in heated water. Combine solutions, adjust volume to 1 L, mix thoroughly, autoclave at 121℃ for 15 min, and pour plates aseptically.
**Sodium Carboxymethylcellulose (CMC-Na) Medium**	CMC-Na 20.0 g, $MgSO_4$ 0.5 g, KH_2PO_4 1 g, NaCl 0.5 g, agar 20.0 g	Prepare CMC-Na solution: Gradually add 20.0 g CMC-Na (viscosity 300–500 mPa·s) to boiling purified water with constant stirring. Separately, dissolve $MgSO_4$ (0.5 g), KH_2PO_4 (1.0 g), NaCl (0.5 g), and agar (20.0 g) in heated water. Combine solutions, adjust to 1 L, mix thor-oughly, autoclave at 121℃ for 15 min, and pour plates aseptically
**Skim Milk Medium**	skim milk 3.0g, NaCl 0.5 g agar 2.0 g	Configure A liquid and B liquid respectively, Solution A: Suspend 3.0 g skim milk powder in 100 mL water. Solution B: Dissolve NaCl (0.5 g) and agar (2.0 g) in 100 mL water with heating. Autoclave both solutions at 121℃ for 15 min, then mix aseptically and pour plates.

Continued Table S1. Preparation of Medium

Types of culture media	Components of the medium	Preparation method
**Nutrient agar (NA, g·L^{-1})**	Peptone 10.0 g, beef extract 5.0 g, NaCl 5.0 g, agar 15.0 g	Peptone 10.0 g, beef extract 5.0 g, NaCl 5.0 g, agar 15.0 g, adjust pH to 7.0 – 7.2. Liquid medium (nutrient broth, NB) is prepared using the same formulation but without agar, and is used for culturing bacteria
**PDB Liquid Medium**	Potato extract 6.0 g, Dextrose 20.0 g	Suspend 26.0 g of Potato Dextrose Broth (PDB) powder in 1,000 mL distilled water. Dissolve completely by stirring, dispense into appropri-ate vessels, and autoclave at 121℃ (103.4 kPa) for 15 min for subsequent use
**Sodium Carboxymethylcellulose (CMC-Na) Liquid Medium**	CMC-Na 10.0 g, $NaNO_3$ 2.0 g, K_2HPO_4 1.0 g, KCl 0.5 g, $MgSO_4·7H_2O$ 0.5 g, $FeSO_4$ 0.01 g, pH 9.5	Dissolve 14.01 g of CMC-Na medium powder in 1,000 mL distilled water with heating (60 ℃) and continuous stirring. Dispense and autoclave at 121℃ for 15 min for subsequent use.
**Inorganic Salt Starch & Skim Milk Liquid Media**	——	Prepared according to the methods described in ‘Inorganic Salt Starch Medium & Skim Milk Medium’ for their respective solid media formulations, with omission of agar.

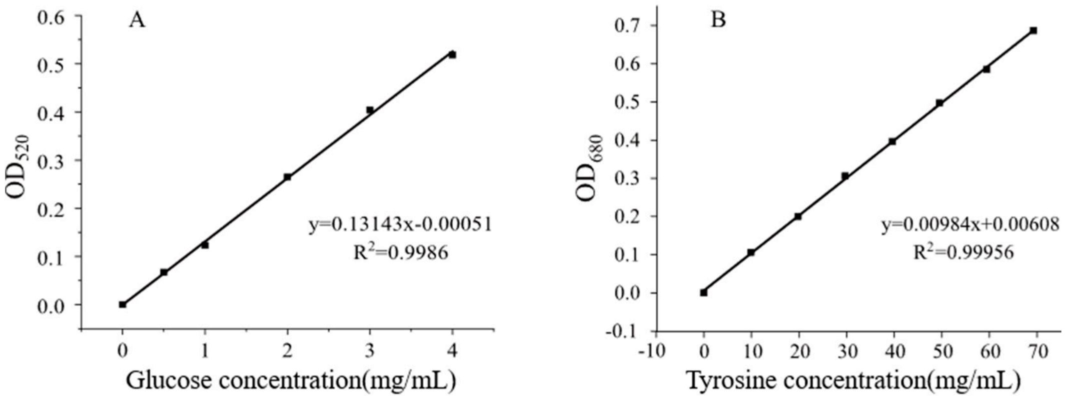

Figure S17. Standard curve of determination of content glucose(A) and tyrosine (B).
